# Examination of Adsorption Orientation of Amyloidogenic Peptides Over Nano-Gold Colloidal Particle Surfaces

**DOI:** 10.3390/ijms20215354

**Published:** 2019-10-28

**Authors:** Kazushige Yokoyama, Kieran Brown, Peter Shevlin, Jack Jenkins, Elizabeth D’Ambrosio, Nicole Ralbovsky, Jessica Battaglia, Ishan Deshmukh, Akane Ichiki

**Affiliations:** Department of Chemistry, The State University of New York at Geneseo College, Geneseo, NY 14454, USA; kb6627500@gmail.com (K.B.); PeterJShevlin@gmail.com (P.S.); jackjenk@iu.edu (J.J.); edambros@udel.edu (E.D.); nralbovsky@albany.edu (N.R.); jessicabattaglia120@gmail.com (J.B.); isd1@geneseo.edu (I.D.); ai7@geneseo.edu (A.I.)

**Keywords:** amyloidogenic peptides, amyloid beta, alpha synuclein, beta 2 microglobulin, nano-gold colloids, peptide coverage, aggregation, adsorption orientation, spiking-out orientation, gyration

## Abstract

The adsorption of amyloidogenic peptides, amyloid beta 1–40 (Aβ_1–40_), alpha-synuclein (α-syn), and beta 2 microglobulin (β2m), was attempted over the surface of nano-gold colloidal particles, ranging from d = 10 to 100 nm in diameter (*d*). The spectroscopic inspection between pH 2 and pH 12 successfully extracted the critical pH point (pH_o_) at which the color change of the amyloidogenic peptide-coated nano-gold colloids occurred due to aggregation of the nano-gold colloids. The change in surface property caused by the degree of peptide coverage was hypothesized to reflect the ΔpH_o_, which is the difference in pH_o_ between bare gold colloids and peptide coated gold colloids. The coverage ratio (Θ) for all amyloidogenic peptides over gold colloid of different sizes was extracted by assuming Θ = 0 at ΔpH_o_ = 0. Remarkably, Θ was found to have a nano-gold colloidal size dependence, however, this nano-size dependence was not simply correlated with *d*. The geometric analysis and simulation of reproducing Θ was conducted by assuming a prolate shape of all amyloidogenic peptides. The simulation concluded that a spiking-out orientation of a prolate was required in order to reproduce the extracted Θ. The involvement of a secondary layer was suggested; this secondary layer was considered to be due to the networking of the peptides. An extracted average distance of networking between adjacent gold colloids supports the binding of peptides as if they are “entangled” and enclosed in an interfacial distance that was found to be approximately 2 nm. The complex nano-size dependence of Θ was explained by available spacing between adjacent prolates. When the secondary layer was formed, Aβ_1–40_ and α-syn possessed a higher affinity to a partially negative nano-gold colloidal surface. However, β2m peptides tend to interact with each other. This difference was explained by the difference in partial charge distribution over a monomer. Both Aβ_1–40_ and α-syn are considered to have a partial charge (especially δ+) distribution centering around the prolate axis. The β2m, however, possesses a distorted charge distribution. For a lower Θ (i.e., Θ <0.5), a prolate was assumed to conduct a gyration motion, maintaining the spiking-out orientation to fill in the unoccupied space with a tilting angle ranging between 5° and 58° depending on the nano-scale and peptide coated to the gold colloid.

## 1. Introduction

The interface of a solid surface and a protein at the nanoscale level are of interests for many applications including material science, biomedical science (e.g., implantation of artificial bones, heart, organs or blood clotting), industry (e.g., the manufacturing of biosensors, bio separation processes, and drug delivery), and research in the development of new materials. The functionalities of peptide coated nanomaterials have remarkably broad applications in areas where nano-size has a very significant effect [1], including nano-light switching devices [2], disease controlling materials combined with DNA [3], DNA sensors [4], control of human cell activity [5], photo dynamic therapy [6], and optical biosensors that quantify heavy metal pollution in water [7]. However, very little is known regarding how the proteins adhere to nanoscale solid surfaces.

Amyloidogenic peptides, such as Aβ_1–40_ or Aβ_1–42_, α-synuclein (α-syn), and β2 microglobulin (β2m) are all regarded as hallmark peptides associated with key onset mechanisms of neurodegenerative diseases, such as Alzheimer’s disease or Parkinson’s disease. Because of this, the formation process and characterization of amyloid fibrils have been extensively investigated. Fibrils are usually several hundreds of micrometers in size and consist of pre-fibrils, which are built of unit oligomers. Therefore, the formation of unit oligomers from soluble and nontoxic monomers is regarded as a key intermediate process of fibrillogenesis and is considered to be a reversible process. On the other hand, the formation of fibrils or pre-fibrils are considered non-reversible processes. The nuclei-based pre-fibril formation mechanism is considered to be the most reasonable method for interpreting the fibril formation process. A key stage in this fibrillogenesis is the formation of an intermediate oligomer through a reversible process which then leads to one-direction pre-fibril formation. A major conformational change of the monomer to an intermediate oligomer requires significant protein folding, requiring a Gibbs energy of −10 kcal/mol [8,9,10] based on computational calculations. The most important on-set process of any fibrillogenesis is networking between peptides. Considering that fibrils are formed irreversibly, the networking between peptides must be “effective” and occur due to a strong interaction. However, a direct investigation of this networking process is still lacking.

The PI’s group has been investigating the reversible self-assembly process (i.e., reversible networking process) on amyloidogenic peptide-coated gold colloidal nanoparticles. The peptides are relatively small, amphiphilic (i.e., consists of both hydrophilic and hydrophobic segments) peptides and the temperature/pH conditions for folded/unfolded conformations are well studied. The great advantage of this system is that a monomer peptide can be prepared on the nano-surface by orientating each peptide so that it may undergo the most effective networking process. Peptides are adsorbed over the nano-surface and are used to make connections between two nano-particle surfaces by making networks between peptides. Because of the networking between peptides, the assembly to the gold colloidal aggregates results in a drastic change in a spectroscopic feature, meaning that the networking process can thus be spectroscopically probed. Therefore, this work is viewed as the best prototype system to learn how nanoscale surface potentials interact with a peptide and if a specific structure can be selectively constructed [11,12,13]. Although these peptides eventually form irreversible insoluble amyloid fibrils, initial stages in fibrillogenesis are still reversible processes. We hypothesize that the peptide-peptide networking must be established by an unfolded conformation of each peptide and this unfolded conformation will be strongly enhanced at the nano-particle surface. As observed in negatively charged micelles and Teflon particles, β-sheet formation of Aβ on hydrophobic graphite surfaces [14] or at air–water interfaces [15] indicate an involvement of interfacial surface potential utilized for the conforming intermediate [16,17,18,19,20].

This study aims to clarify (1) an exact attaching sequence or portion of the peptide and (2) orientation of the peptide over the nano-scale surface, and (3) identify the probable conformation of peptides for successful networking. It is well known that the amyloidogenic peptides (e.g., amyloid beta: Aβ; beta 2 microglobulin: β2m; and alpha-synuclein: α-syn) adsorb onto a gold surface through a sulfur atom of a thiol (–SH) group. These amyloidogenic peptides undergo drastic structural changes (protein folding) to form many units of toxic polymers that eventually combine to create a few micron-sized fibers (i.e., amyloid fibrils), which are known to cause neurodegenerative diseases [21,22,23,24,25,26,27,28,29,30,31,32,33,34,35]. However, Aβ and α-syn do not possess any sequences which contain a thiol group (i.e., Cysteine (Cys, C)). Contradicting the lack of a Cys sequence, the existence of gold nano-colloids were reported to enhance peptide-peptide networking using Aβ adsorbed on a nano-gold colloid as a “core” of a fiber [36].

There is no clear explanation of why amyloidogenic peptides adsorb onto gold so effectively. Since the detailed structural information of adsorbed peptides at the “core” is not known, contributing factors to the peptide-peptide networking needs to be fully investigated. This study describes a systematic method used to extract both a plausible peptide orientation and which segments interact with the colloidal surface. Based on these data, in Section 4.3.3 we describe a novel systematic procedure to extract the coverage ratio of peptide (Θ) onto the nano-gold colloids. Quite unexpectedly, the surface coverage conditions appeared to depend somewhat on the nano-particle size. While simulation does not explain the nano-size dependence on surface coverage, the observed trend suggests a plausible “packing” formation of the peptide due to a physical surface area condition.

## 2. Results

### 2.1. Extraction of Θ

**Figure 1:**
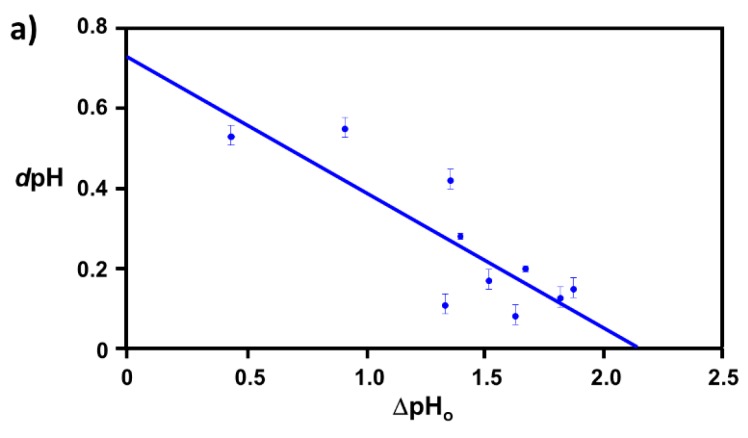

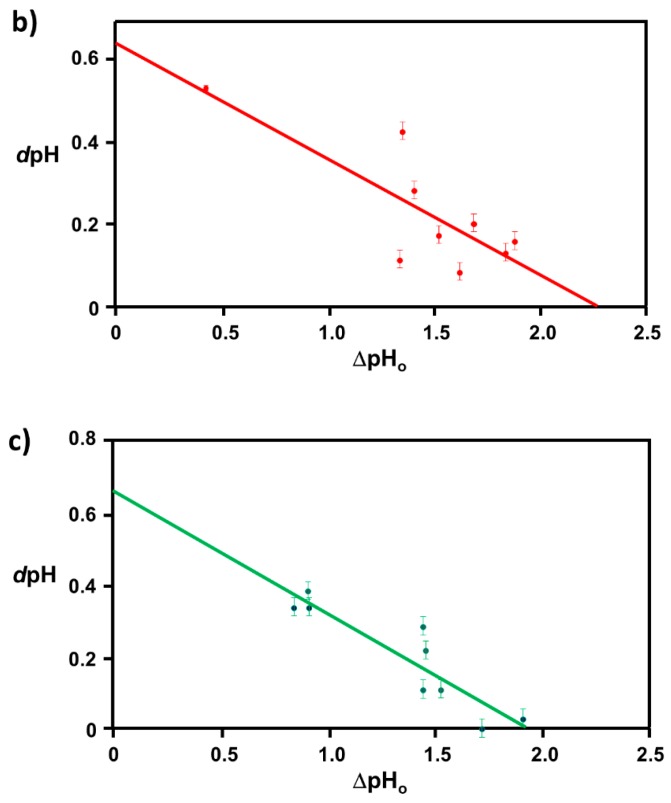
A hypothesized linear relationship between *d*pH vs. ΔpH_o_ was plotted for each amyloidogenic peptide-coated nano gold colloid, (**a**). Aβ_1–40_ in blue, (**b**). α-syn in red, and (**c**).β2m in green based on the values obtained from fitting sigmoidal plot with Equation (1). Each linear line was given as a guide for a correlation between *d*pH vs. ΔpH_o_. (*d*pH = m ΔpH_o_ + b). Supporting information of this figure is available at Supplementary Materials.

This hypothesis stating a linear relationship between dpH and ΔpH_o_ was clearly proved to be true when *d*pH was plotted as a function of ΔpH_o_ for each tested gold colloidal size and in all three amyloidogenic peptides (Aβ_1–40_, α-syn, and β2m) as shown in Figure 1, while ovalbumin-coated gold colloid did not show any sign of linear relationship [36]. Each data point shown in Figure 1 corresponds to different gold colloidal sizes for a given peptide. It shows that the coverage area is determined by the size of the nano-particle and there must be an equilibrium electrostatic shielding value for a given nano-gold metal surface. The average coverage ratio, Θ_avg._, for each amyloidogenic peptide was extracted as: Θ_avg_ (Aβ_1–40_) = 0.6 ± 0.2, Θ_avg_ (α-syn) = 0.6 ± 0.2, and Θ_avg_ (β2m) = 0.7 ± 0.2. By using the reported structural data most suited to the conditions of our work, the axial length *a* and *b* (*a* < *b*) of an approximated prolate for Aβ_1–40_ [17] α-syn [37], and β2m [38] were initially estimated to be: Aβ_1–40_ (*a*, *b*) = (2.1 nm, 4.1 nm), α-syn (*a*, *b*) = (3.1 nm, 8.5 nm), and β2m (*a*, *b*) = (2.5 nm, 4.6 nm).

### 2.2. Distance between Colloidal Particles

**Figure 2:**
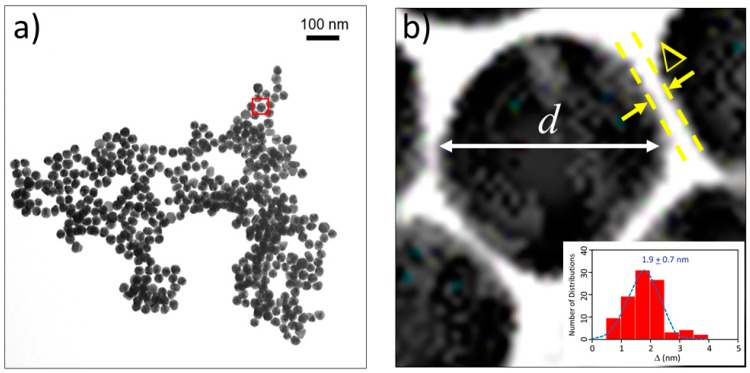
(**a**) A TEM (transmission electron microscopy) image of 30 nm gold colloidal particles coated with β2m at pH 4. The scale bar for 100 nm is shown as a guide. (**b**) A magnified section of the red box showing the diameter of a gold colloid *d* and the distance between the adjacent colloidal particles (Δ). (Inset): A typical histogram showing the Δ and the observed numbers of the distribution. The distribution was fit by a Gaussian profile and shown by a dotted curve. This histogram is for the β2m-coated 30 nm gold colloid.

A representative TEM image of β2m-coated 30 nm gold colloids is shown in Figure 2a. As the magnified image clearly shows, distinct spacing noted as “Δ” between gold colloids were observed. (Figure 2b) The spaces between the gold particles was measured over multiple measurements per image for each size of gold particle and β2m. The morphology of the gold colloidal aggregates coated with Aβ_1–40_, α-syn, as well as albumin were also studied, and the aggregates formed were more extensive in size and number (size exceeded up to a few microns and the number of gold colloids far exceeded 500 or 1000) than those formed by β2m-coated gold colloid. Therefore, the density of the aggregates by Aβ_1–40,_ as well as albumin coated gold colloid aggregates, extended in the longitudinal direction resulting in preventing the view the section of planar topology. In contrast, β2m formed relatively smaller aggregates, ranging within 500 nm with less than 100 colloids. This allowed us to visualize a two-dimensional view of the aggregate and made it possible to disclose the spacing between two adjacent gold colloids. While the peptide character of the studied peptides is not equivalent, we assume the networking character can be similar. Also, the physical dimension of the networking section compared to the diameter of each colloid can be approximated to be the same. Therefore, the information obtained for β2m will be shown as illustrative for the other two peptide coated gold colloids.

The average distances of adjacent nano-gold colloids and the average number of gold nano-particles to form one aggregate for *d* = 10, 30, 60, and 80 nm particles are summarized in Table 1. We can conclude that the average distance of the adjacent nano-gold colloids was extracted to be 2.0 ± 0.8 nm, indicating insignificant size dependence of the gold colloid and the number of nano-gold colloids forming a cluster.

### 2.3. Simulation of Θ and Orientation

**Figure 3:**
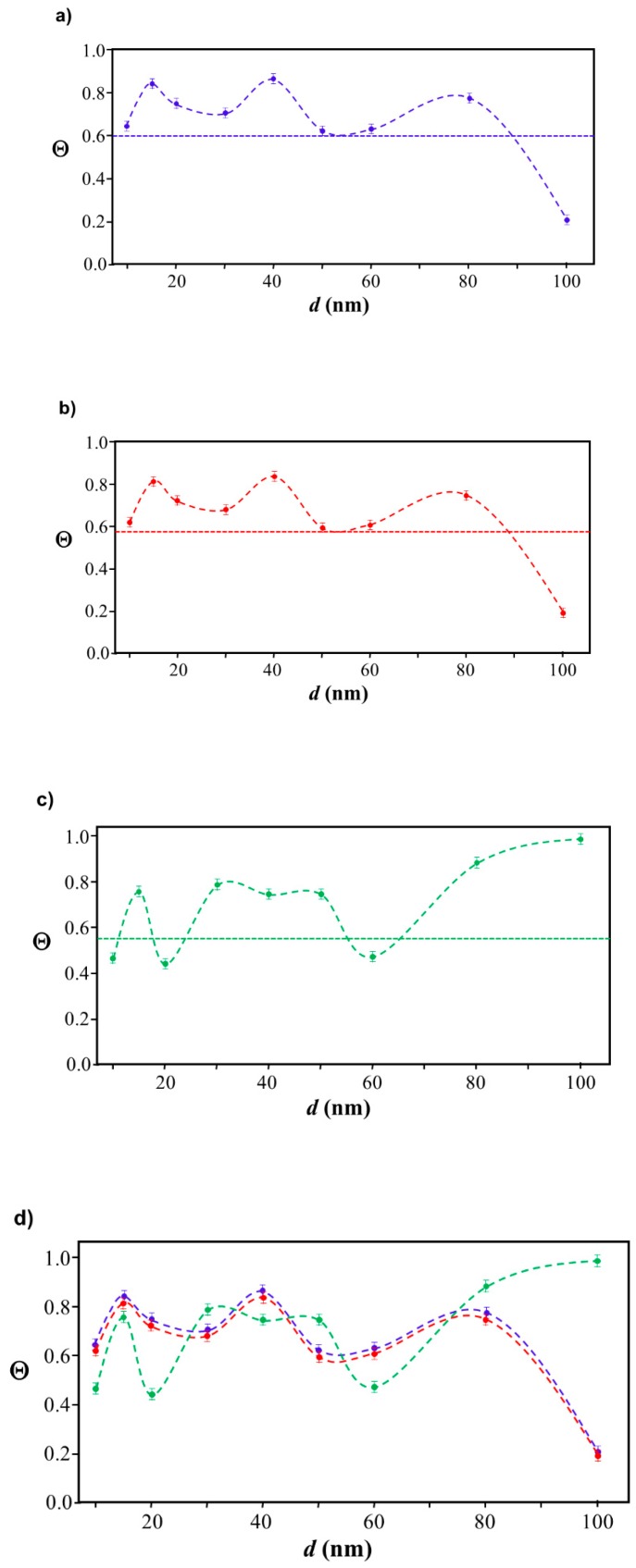
A plot for experimentally obtained Θ for (**a**) Aβ_1–40_ in blue (**b**) α-syn in red, and (**c**) β2m in green as a function of reported gold diameter *d* (nm). All data points were extracted by using Equation (2). Each dashed curve line is simulated by a method described in Section 4.3.3 with the parameters tabulate in Table 2. The dotted line shows an upper limit of the Θ value obtained by a single layer model. (**d**) The over-laid plots of all (**a**–**c**) are shown, and indicating (**a**,**b**) almost overlay each other.

The procedures explained in Section 4.3.3 was applied to analyze the data points shown in Figure 3. The optimized axial lengths of prolate for three amyloidogenic peptides as a function of colloidal size are listed in Table 2. In order to reproduce extracted Θ, the spiking-out orientation need to be utilized for all cases except for *d* = 100 nm gold coated with Aβ_1–40_ and α-syn as well as *d* = 10, 20, 60 nm gold coated with β2m, which exhibited a lie-down orientation. For example, Aβ_1–40_ coated gold 20 nm diameter showed Θ_obs_ = 0.74 for *a* = 1.4 nm and *b* = 2.2 nm in order to reach maximum coverage ratio 0.37 under the first layer, Θ*_f_*_,cal_, with total number of attached peptides to be *n*_total_ = 111. After the second layer was added, it is calculated to have a maximum of Θ*_s_*_,cal_ = 0.72. In order to be consistent with the observed Θ_obs_ = 0.74, a contribution of the second layer, γ (Θ*_s_*_,cal_) should be 0.51, so that Θ_obs_ = 0.74 = Θ*_f_*_,cal_ + γ (Θ*_s_*_,cal_) × Θ*_s_*_,cal_ =0.37 + 0.51 × 0.72=0.37 + 0.37. There was almost no contribution of the second layer when Θ_obs_ < 0.5 for *d* = 100 nm of both Aβ_1–40_ and α-syn as well as *d* = 10, 20, and 60 nm for β2m.

**Figure 4:**
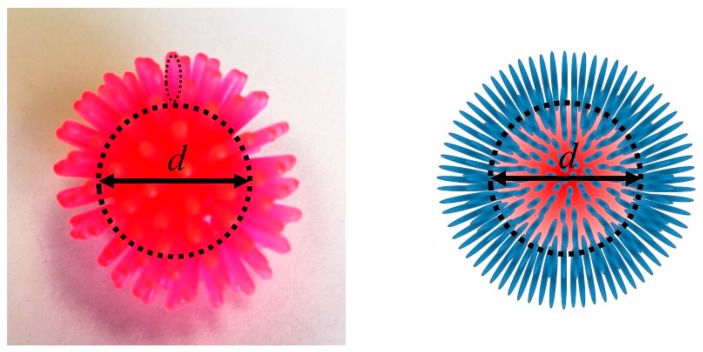
A picture of an illustrative model and sketch demonstrating a peptide aligning over the surface of a gold colloidal particle with a diameter, *d*. In the image on the **left**, the peptide and gold core are both shown with the same color, and a dotted oval indicates a prolate shaped peptide. In the sketch on the **right**, prolate shaped peptide is shown in blue and the core gold colloid with diameter d is shown in red sphere.

In summary, as shown in Figure 4, all studied amyloidogenic peptides interacted with nano-gold surface at the original pH where a sample was prepared (~pH 7) with unfolded condition with spiking out condition.

## 3. Discussions

### 3.1. Elucidation of Θ and Spectroscopic Measurement

The spectroscopic measurement used in this study exhibits the transition of dispersed peptide-coated gold colloid to aggregated gold colloid through a networking of peptides on the colloidal surface. The surface charge or surface charge potential of each colloid should ideally be neutral, in order to avoid mutual repulsions which would impede aggregation. It is speculated that an aggregation of Aβ_1–40_ coated gold colloid takes place at an isoelectric point, pI, of Aβ_1–40_ (pI = 5.2) [39]. If so, a constant pH_o_ (~pI) is expected to be observed regardless of the sizes of gold colloid. However the observed pH_o_ for Aβ_1–40_ coated gold colloid ranging from *d* = 10 nm to *d* = 100 nm scanned between pH_o_ = 4.38 ± 0.06 for *d* = 100 nm and pH_o_ = 6.20 ± 0.01 for *d* = 40 nm [40]. The extracted pH_o_ implies the amount of positive charge, i.e., [H_3_O^+^], required to neutralize the colloidal surface. This work demonstrated the correlation between ΔpH_o_ and *d*pH as a key concept in extracting the change of surface charge potential of the gold colloidal particle. Due to the fact that the bare gold colloid possesses surface plasmon (electrons) over its surface, the excess amount of [H_3_O^+^] needs to be supplied in order to neutralize the surface. Thus, the ΔpH_o_ shows the difference of the amount of [H_3_O^+^] required between bare gold colloid and the peptide-coated gold colloid. Essentially, this means that the ΔpH_o_ indirectly shows the amount of the negative charges quenched due to the attachment of the peptides over the surface and the changing surface charge potential of the colloidal surface.

The measured quantity *d*pH is defined in a method described in Section 4.3.1. Since λ_peak_^(1)^ is the first derivative of λ_peak_(pH), it has the dimension of Δλ/ΔpH (Δλ indicates the wavelength change associated to the transition from a dispersed stage to an aggregated stage, and ΔpH indicates the amount of H_3_O^+^ ion needed for that transition). The marking wavelengths λ_max_ and λ_min_ indicate that the colloid is at the aggregated stage (λ_max_) or at the dispersed stage (λ_min_). In practice, the marking wavelengths can be treated as dimensionless numbers or as an index (or with arbitrary units), with the replacement of λ_peak_^(1)^ by ΔΛ/ΔpH, where ΔΛ implies the difference of an index Λ. Overall, *d*pH indicates an inverse of λ_peak_^(1)^ and it is proportional to ΔpH, if ΔΛ is treated as a constant. Therefore, because ΔpH ∝ –log Ω and *d*pH ∝ log Ω, where Ω is a constant associated with charge. Thus, in principle, there should be a linear correlation between *d*pH and ΔpH.

A. Wang et. al. reported a pH dependence in protein coverage [41], and it is presumable that the surface charge condition is fully influenced by the residual pH condition. This suggests that the peptides start occupying and aggregating at the gold surface as the pH point gets closer to pH_o_. If this is the case, peptides may not adsorb on the gold surface with our reported Θ values at pH > pH_o_. However, our work is designed to determine pH_o_, where the coverage of amyloidogenic peptide was already completed, our approach allows us to extract Θ only at pH_o_, and it limits a quantitative conclusion regarding the pH dependence of Θ.

### 3.2. Orientation of the Peptide over the Surface of Gold Colloidal Surface

**Figure 5:**
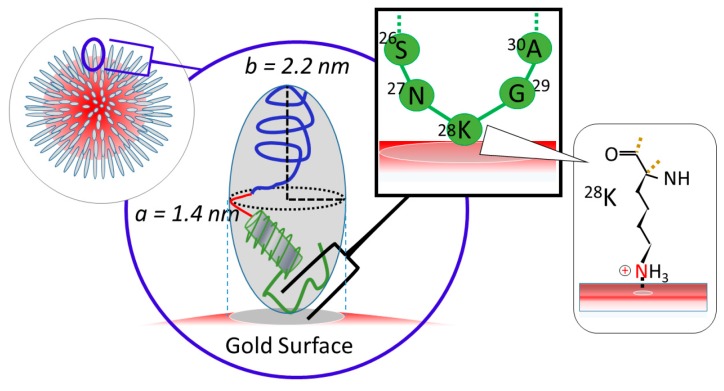
The proposed attachment structure of Aβ_1–40_ over the surface of a gold colloidal particle. At the left top sketch shows the proposed peptide orientation adsorbed over gold nano particle. In the middle the blow up of each peptide with a prolate shape is shown. Inside the prolate, a sketch of Aβ_1–40_ is shown within a prolate of *a* = 1.4 nm and *b* = 2.2 nm. On the top, a blow up and hypothesis of sequences responsible for the adsorption on the gold surface are shown and ^28^K was speculated to be in a direct contact with gold surface. At the further right, a structure of Lysine (K) is shown and -NH_3_^+^ group is estimated to be a central point for an adsorption.

Relatively high Θ (i.e., Θ >0.5) can be accommodated by filling the surface area with a greater number of smaller unit surface area. Thus, the most supported orientation of the prolates is a spiking-out orientation, as sketched in Figure 5. While a “lie-down” orientation was highly expected to establish more interaction between peptide and the gold surface, the higher coverage was only established by creating a larger amount of smaller contacting areas. As supporting evidence, a very similar orientation was reported by Stellaci’s group for bipolar polymer spiking out of gold nanoparticle sphere [42,43,44,45,46,47,48]. Considering that the gold colloid has a partially negative surface charge, any positively charged sequence can interact with it electrostatically. Since Aβ_1–40_ coated gold colloid is dissolved in an aqueous solution, hydrophobic segments of Aβ_1–40_ (sequences 23–40, C-terminal side) must be used for contacting the gold colloidal surface, causing hydrophilic segments of Aβ_1–40_ (sequences 1–22, N-terminal side) to face outside, making it soluble in water. Among the hydrophobic sequences (23–40), only ^28^Lysine (^28^Lys, ^28^K) can be positively charged at neutral conditions. Therefore, it is hypothesized that –N^+^– part of the ^28^Lysine is responsible for contacting on the gold colloidal surface as shown Figure 5.

Due to more complexities in structure, identification of binding sites for α-syn and β2m were less clear. Even so, a similar approach and rough estimation of the site is possible. For α-syn, residues 61–95, or the so-called NAC (Non-Aβ Component) region [49,50], is highly likely to be the region where the peptide is bound to the nano-gold surface. More specifically, ^80^K, ^96^K, and/or ^97^K are candidate residues that could be responsible for the colloidal attachment.

In a similar way, however, with a more complex situation, β2m is considered to possess hydrophobic (and aromatic-rich) region in residues 62–70 implying ^63^R (^63^Arginine), ^66^K, or ^69^H to be a plausible binding sites to the nano-surface [51]. If we assume multiple concurrent contacting spots are available, the mobility of β2m must be reduced and this can separate the binding property of β2m different from the other two peptides. In order to explain a negative correlation observed in Θ vs. S_d_ plot (Section 3.4), β2m was speculated to be a prolate with negative side facing outward. It is quite likely that the sequence from 50 to 58 are the section responsible for the above-mentioned section since the negatively charged section of ^52^D (^52^Aspartic Acid), ^54^E (^54^Glutamic Acid), and ^56^D are located therein.

The current model used for extracting Θ was tested for chicken ovalbumin as an example of a globular protein. There was no correlation found between *d*pH and ΔpH, and we assume that the model can be applied only for amyloidogenic peptides that clearly exhibits folded and unfolded conformations which are drastically different. Also the section of adsorption site has to be clearly determined no matter which size of the gold colloid was applied, otherwise clear mapping of charge distribution due to peptide (as explained in Section 3.4) cannot be obtained. The prolate shape localizes a partial charge in a relatively smaller region, which can become an adsorption point. In contrast, a globular protein takes a spherical shape creating a broader and more homogeneous partial charge distribution. This results in a less sensitive response in aggregation as a function of pH change, which results in a poorly defined *d*pH value in Equation (1), and reducing a correlation between ΔpH_o_ and *d*pH.

### 3.3. Networking of the Peptide at an Interfacial Area

**Figure 6:**
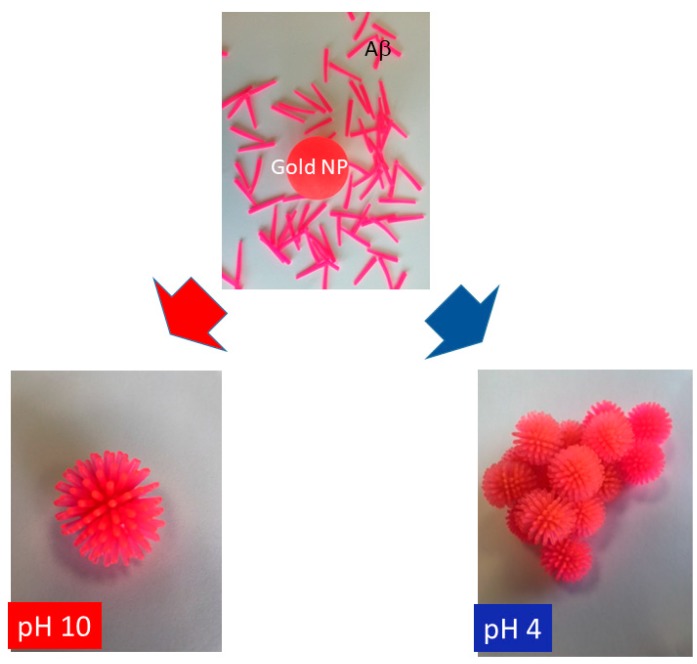
A demonstrative picture of peptides being adsorbed over a surface of gold nano-particle (NP) and adsorption of peptide with spiking out orientation under pH 10 (bottom left) and forming a network with each other in order to form gold colloid aggregates at pH 4 (bottom right). The color of peptide and gold colloid are shown in the same color.

While the aggregation of Aβ_1–40_ coated gold colloid develops highly condensed networking which results in the mutual overlapping of gold colloids in both horizontal and latitudinal directions, the aggregation of β2m-coated gold colloid was less condensed, and TEM images enabled us to observe the spacing between adjacent gold colloids (Δ = 1.9 ± 0.7 nm), particularly around the edge area of the aggregates. Since Aβ_1–40_ and β2m both approximate as prolate tops with similar dimensions, the information on this spacing between adjacent gold colloid can be extrapolated to these two peptide systems. This result implies that the peptide layer covered the gold colloidal surface at a thickness of 0.95 nm [52]. If we consider a monolayer of peptide with spiking-out orientation, the adjacent distance between two gold colloid should correspond to 2*b*. This assumption strongly contradicts the extracted Δ (~2 nm) since calculated 2*b* value are 4.4 nm for Aβ_1–40_, 14.8 nm for α-syn, and 9.2 nm for β2m based on the values shown in Table 2. In order to allow peptides to be constrained within 2 nm spacing, the most probable conformation allows the peptides to be bent or spiraled around each other at the interface. Since amyloidogenic peptides studied in this work are all regarded as disordered proteins, it is reasonable to assume that disordered regions are flexible enough to take best suited configuration including a bent form in order to fit in 2 nm inter-colloidal surfaces. The process of gold colloidal aggregation is summarized as the process of a mixture (interaction) between monomer and gold colloidal surface, followed by the adsorption of each monomer over the nano-colloidal surface, and under acidic pH, the networking of peptides forming the gold colloid aggregation (Figure 6).

Based on the fact that all experimental observation needs to involve a second layer, we deduce that the first layer is responsible for the coverage of nano-gold surface and the second layer is the result of networking to the first layer of each peptide coated gold colloid. Due to the spiking-out orientation of the first layer, this would leave accessible another site for further networking as the peptide conformation becomes unfolded. The networking between dual peptides at an interface matches with a speculation of a dimer formation concluded in our previous work [53].

### 3.4. Verification of the Relationship between Physical Displacement and Coverage Ratio

**Figure 7:**
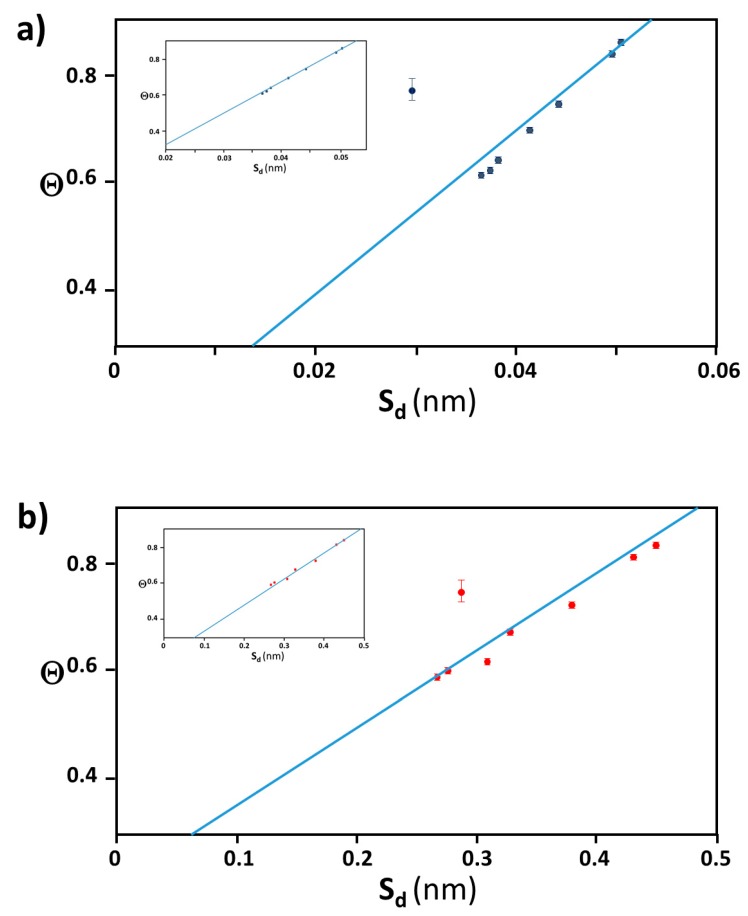

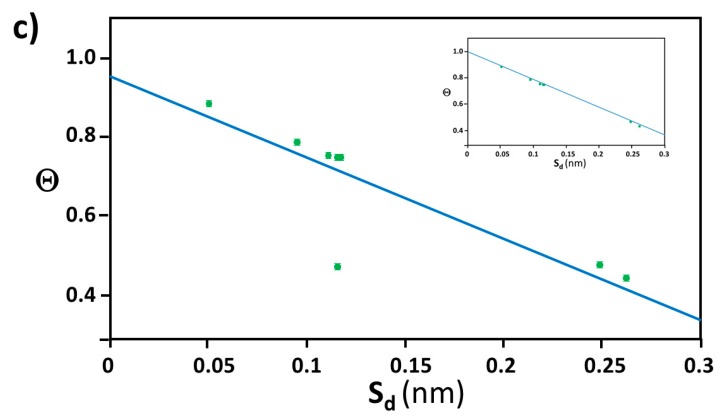
The best optimized plot of Θ vs. S_d_ for (**a**) Aβ_1–40_ in blue, (**b**) α-syn in red, and (**c**) β2m in green, where fitting values for the linear relationship Θ = Φ S_d_ + ε. In each plot, there was always one deviating data point from the linear trend (*d* = 80 nm for Aβ_1–40_ and α-syn, *d* = 60 nm for β2m), and the insets show the plot when each outlier point was removed. Supporting information of this figure is available at Supplementary Materials.

Our first instinct was that Θ was dominated by the molecular interaction between gold surface and peptide’s terminus responsible for an electrostatic interaction. Therefore, the surface field reflecting from the surface curvature would be proportional to molecular interaction and may influence the surface interaction and coverage. However, by assuming that simple term of curvature is proportional to an inverse of radius, we did not see any correlation between a curvature and Θ, as also implied by the complex relationship between Θ and d shown in Figure 4. This implies that at least obvious chemical interaction does not explain the intrinsic reasoning of Θ and its nano-size dependence. While no correlation between the gold colloidal size and its coverage ratio of peptide (Θ) was found in our study, we attempted to find justification of Θ for a given nano-size of gold colloid by using a mathematical approach without involving intermolecular forces. The most simplified explanation of higher or lower coverage is gained from calculating how much space is wasted by a given unit adsorbent. However, the coverage ratio cannot be simply predicted as a function of surface area. For example, if the area to be covered increases, the unit area of an adsorbent may not utilize given space without leaving an unoccupied area, and so the coverage ratio may not increase. The equatorial belt area was used as an index of the effectiveness of space usage, and the spacing between each prolate (S*_d_*) should be correlated with the coverage ratio (Θ). For example, a prolate of Aβ_1–40_ (*a* = 1.4 nm and *b* = 2.2 nm) covering a 40 nm (*d* = 40.6 nm) has Θ = 0.86), and 100 nm (*d* = 99.5 nm)has Θ of 0.20. When maximum prolate with dimension of (*a* = 1.4 nm and *b* = 2.2 nm) was distributed equatorial belt of each gold colloid, S*_d_* = 0.051 nm for 40 nm with n*_eq_* = 50 and S*_d_* = 0.012 nm for 100 nm with n*_eq_* = 175 demonstrating that the larger the S*_d_*, the higher the Θ. A clear correlation between Θ and S*_d_* was confirmed for Aβ_1–40_ and α-syn as shown in Figure 7a,b as a positive slope for β2m as shown in Figure 7c as a negative slope. The finalized fitting parameters and fitting procedures will be further detailed in a report by Yokoyama and Ichiki [54].

**Figure 8:**
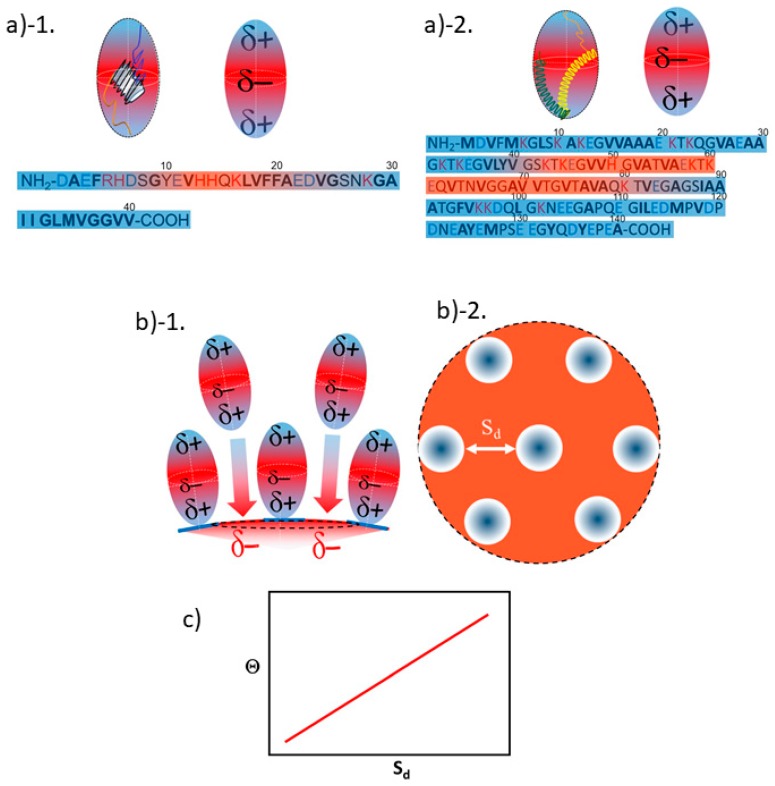
A sketch explaining a positive linear proportionality between Θ vs. S_d_, (**a**) simulation of prolate and charge distribution of Aβ_1–40_ (**(a)-1**) and of β2m (**(a)-2**). The sequences of each peptide are shown with the colored bar indicating δ– (in red) or δ+ (in blue). (**(b)-1**). A side view of a prolate top peptide with a partially positive side (δ+) of dipole attaching to the partially negative surface (δ–) of gold colloid. An extra prolate dipole attracted for the space of δ–, if S_d_ has enough length to let an extra prolate in. (**(b)-2**) The birds eye view of the surface showing area appears as δ– indicated by red is the highly probable are for an extra prolate to be interacted and may lead to an attachment. (**c**). A graph explaining the expected trend between Θ as a function of S_d._

A positive linear relationship between Θ and S*_d_* is explained by considering that both Aβ_1–40_ (Figure 8(a-1)) and α-syn (Figure 8(a-2)) are simplified as a prolate with δ+ region at the adsorption side and opposite side (i.e., exposing side to the outward) as sketched in Figure 8(b-1), respectively. As the prolate attaches onto the gold surface through the δ+ region of a prolate, it also creates the δ+ region on the gold surface as indicated in Figure 8(b-1,b-2). So that an extra peptides are more invited for adsorption as the gold surface possesses more δ– region when S*_d_* is longer. On the other hand, if S*_d_* is relatively small, not enough δ– region is available for further adsorption of peptides causing the Θ decreased resulting in the positive linear relationship between Θ and S*_d_* (Figure 8c). As it was speculated before, Aβ_1–40_ may be adsorbing on to the surface through ^28^K and α-syn adsorbs on to the surface through ^80^K or ^96^K^97^K. Since those sites are located at relatively close to the N-terminal, it is speculated that δ+ portion of C-terminal side must be exposing outward and away from the colloid surface. As for Aβ_1–40_, we speculate that ^5^R^6^H, ^13^H^14^H, or ^16^K are responsible for distributing δ+ region. The speculated region with δ+ and δ– are indicated by color coded areas in a prolate and bars in sequences as δ+ in blue and δ– in red, respectively (Figure 8(a-1)) As for α-syn, all lysines in the C-terminal region (i.e., ^6^K, ^10^K, ^12^K, ^21^K, ^23^K, ^32^K and ^34^K) are speculated to be exposing toward the outside and away from the colloidal surface side. While much more information is needed, crude estimation of charge distribution was shown in Figure 8(a-2). In a similar manner as shown in Figure 8(a-1), region with δ+ and δ– are indicated by color coded areas in a prolate and bars in sequences as δ+ in blue and δ– in red, respectively.

**Figure 9:**
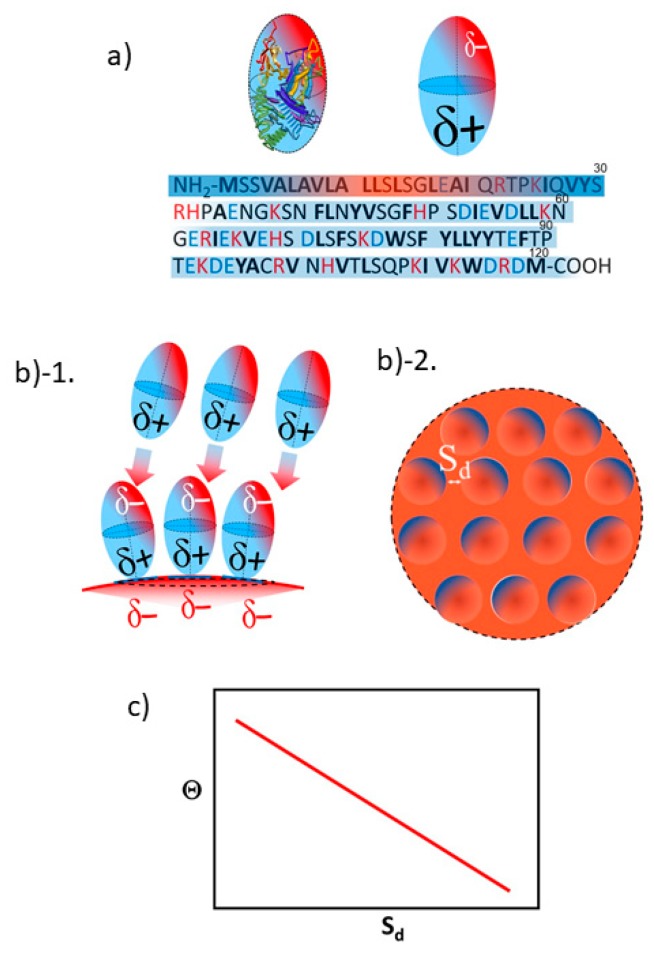
A sketch explaining negative linear relationship between Θ vs. S_d_. (**a**) simulation of prolate and charge distribution of β2m. The sequences are shown with the colored bar indicating δ– (in red) or δ+ (in blue). (**(b)-1**). A side view of a prolate top peptide with δ+ side of dipole attaching to δ– surface of gold colloid. Because a distribution of δ– is expected to be spread from the top to the side toward outside, extra prolate is more attracted as more area of δ– is available. (**(b)-2**). A top view of a focused region in b)-1, where the area appears as δ– as the prolate locate close by shortening of S_d_. (**c**). A graph explaining the expected trend between Θ as a function of S_d,_.

Opposed to what we observed in Aβ_1–40_ and α-syn, β2m exhibited a negative linear slope for Θ vs. S_d_ plot. (Figure 7c). This is interpreted that as each β2m (sketched in Figure 9(b-1)) adsorbs onto the gold surface with δ+ segment as exposing more δ– area to the other side of gold surface as sketched in Figure 9(b-1,b-2). Thus, as S_d_ decreases, it creates a greater effective attraction to the extra β2m resulting in more coverage (i.e., a negative slope for Θ vs. S_d_ plot) as shown in Figure 9c. Since the adsorption site of β2m can be speculated to be at relatively toward the C-terminal side (i.e., ^63^R, ^66^K, or ^69^H), the exposing side away from the gold surface is speculated to be N-terminal side. Thus, it is estimated that^18^E is responsible for providing δ– region. In Figure 9a, region of δ+ and δ– are indicated by color coded areas in a prolate and bars in sequences as δ+ in blue and δ– in red, respectively.

In all three cases explained above, we claim that the spiking-out orientation of the first layer established a corresponding charge distribution seen in each peptide coated gold colloid. If the orientation was lie-down orientation, enhancement of self-adsorption would not take place. For example, a lie-down orientation of prolate dipole in the case of Aβ_1–40_ and α-syn would exhibit a significant amount of δ+ region and not effectively squeeze the prolate dipole with the same orientation. As for the case of β2m, the lie-down orientation exposes a greater amount of δ– region, resulting in a significant repulsion for the peptide attempts to adsorb with the same lie-down orientation.

### 3.5. Justification of Lower Coverage Ratio and Associated Prolate Shape

**Figure 10:**
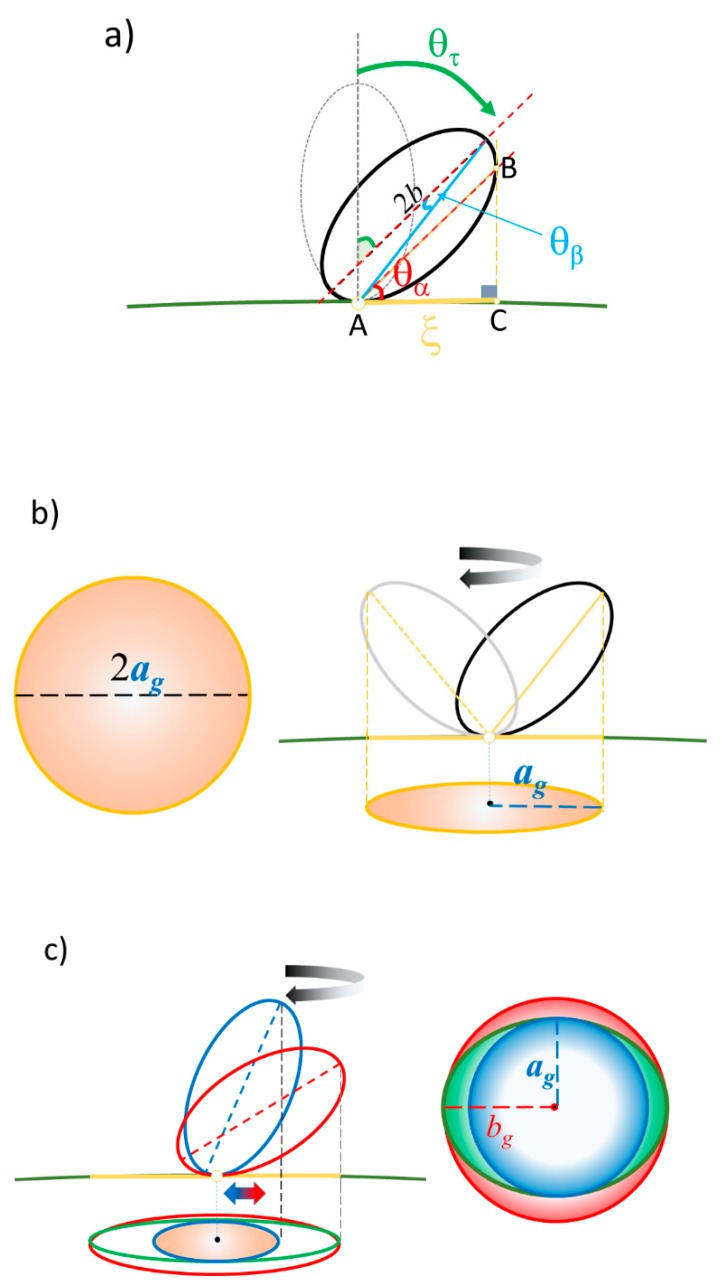
A sketch of the side view of a rotating prolate. (**a**) The tilting of a prolate over the nano-gold surface and approximation for radius ($\bar{AC}$) of the circular plane over the nano surface. Here, θ_α_ is a tangential angle between prolate axis and the surface line, and the tilting angle of a prolate against surface plane is given by an angle θ_τ_. (**b**) A rotational motion of a prolate with a fixed contacting point, resulting in a circular occupied space over the surface. (**c**) A gyration motion of a prolate with a movable contacting point and tilting angle θ_τ_, resulting in an oval (in green) occupied space with axial length of *a*_g_ (blue circle) and *b*_g_ (red circle).

While the overall characteristic of the coverage of amyloidogenic peptides was relatively higher value (i.e., Θ ≥ 0.6), there were only five cases when Θ was < 0.5; Aβ_1–40_ coated *d* = 100 nm gold, α-syn coated *d* = 100 nm gold, β2m-coated *d* = 10, 20, and 60 nm gold. For all cases, the fit was not optimized with a prolate with spiking-out orientation but with lie-down orientation. Under the estimation that the spiking-out orientation is the best orientation to satisfy coverage stability (i.e., effective packing of the surface) and consistent with most of the coverage orientations observed in this experiment. Thus, it is hypothesized that each prolate takes a spiking-out orientation but tilts over the nano-gold surface as shown in Figure 10a and can rotate around the contact point on the nano-gold surface (Figure 10b) resulting in an occupied area with oblate shape as if it takes a lie-down orientation. In order to explain an oval shape occupying over the surface, a gyration type of motion is considered. So that the contact point of the prolate changes due to a change of tilting angle as it rotates over the surface, is plausible (Figure 10c).

From the geometry shown in Figure 10a, $\bar{AC}=2bcos\theta_{\alpha }\theta_{\beta }$, where θ_α_ and θ_β_ are the inner angles as shown in Figure 10a and the length $\bar{AC}$ was approximated as $\bar{AB}\approx 2bcos\theta_{\beta }$ because θ_β_ << 1. A tilting angle of a prolate, θ_τ_ = 90° – θ_α_. The extracted θ_α_ and θ_β_ are listed in Table 3.

**Figure 11:**
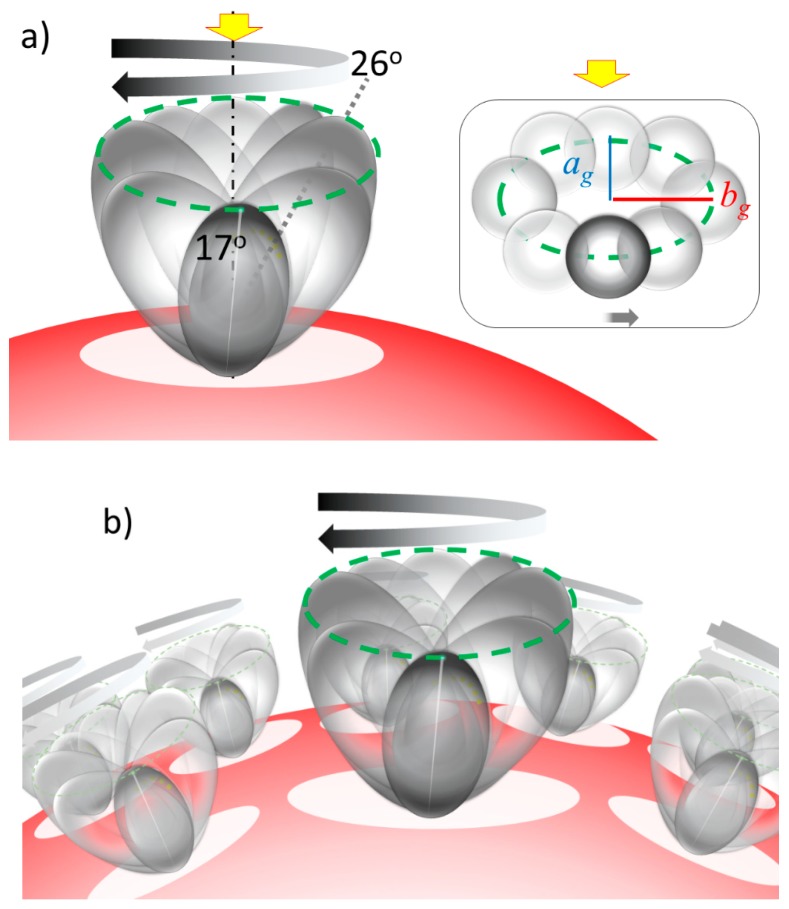
(**a**) The sketch showing the gyration motion of a prolate (*a* = 2.7 nm and *b* = 4.0 nm) representing β2m over a gold nano-particle with a diameter of *d* = 10 nm, where the prolate major axis tilts between 26° and 17° as it rotates over the surface. It results in an oval occupied space with *a_g_* = 2.7 nm and *b_g_* = 4.0 nm. (See Table 3) (**b**) The sketch of a gyrating prolate over the nano-gold particle surface.

An example of extracted gyration motion was demonstrated and sketched in Figure 11 for the case of β2m adsorbed over *d* = 10 nm gold colloid (*d* = 9.80 nm). Focusing on one unit of prolate as shown in Figure 11a, the tilting angle, θ_τ_, changes between 26° and 17° as it rotates, which modulates the surface area. The gyration of the prolate should be taking place simultaneously with the other prolates on the same surface as shown in Figure 11b. We cannot, however, deny that a stationary peptide in an unfolded conformation could occupy the space of the same size calculated by gyration motion. There is a possibility of that the adsorption is more randomized and is an ensemble of multiple orientations. For example, J. A. Yang and et. al., reported that α-syn adsorbs on the poly (allylamide hydrochloride) coated gold nanoparticles with random orientation with an increase in β-sheet and decrease in α-helix structure [55].

## 4. Materials and Methods

### 4.1. Materials

Lyophilized powder of Aβ_1–40_ peptide (MW; 4.2 kDa, 98% HPLC purity) and α-syn (MW: 14.4 kDa, purity >95% by SDS-PAGE) were purchased from r-Peptide (Bogart, GA, USA). Aqueous 220 μM stock solution of Aβ_1–40_ and 64.2 μM stock solution of α-syn were stored at –80 °C. The β2m (MW: 12 kDa/mol, purity >40% by SDS-PAGE) was purchased from AbD Serotec (Raleigh, NC, USA), and aqueous 77.0 μM stock solution was stored at –20 °C. Gold nanoparticles were purchased from Ted Pella, Inc. (Redding, CA, USA) and have the following estimated diameters (d), reported diameter (*d*), and particles per mL in O.D. (Optical Density, ϑ) where ϑ = 0.2 at 528 nm: d = 10 nm (*d* = 9.8 ± 1.0 nm, ϑ = 1.4 × 10^12^ particles mL^−1^), d = 15 nm (*d* = 15.2 ± 1.5 nm, ϑ = 2.8 × 10^11^ particles mL^−1^), d = 20 nm (*d* = 19.7 ± 1.1 nm, ϑ = 1.4 × 10^11^ particles mL^−1^), d = 30 nm (*d* = 30.7 ± 1.3 nm, ϑ = 4.0 × 10^10^ particles mL^−1^), d = 40 nm (*d* = 40.6 ± 1.1 nm, ϑ = 1.8 × 10^10^ particles mL^−1^), d = 50 nm (*d* = 51.5 ± 4 nm, ϑ = 8.2 × 10^9^ particles mL^−1^), d = 60 nm (*d* = 60 ± 1.0 nm, ϑ = 4.3 × 10^9^ particles mL^−1^), d = 80 nm (*d* = 80 ± 1.0 nm, ϑ = 2.2 × 10^9^ particles mL^−1^), and d = 100 nm (*d* = 99.5 ± 1.3 nm, ϑ = 1.6 × 10^9^ particles mL^−1^). The residual components in each colloidal particle can be regarded as identical. In order to maintain stability of the nano-gold colloids against salts, deionized and distilled water were used to prepare all aqueous solutions. All sizes of gold colloids were formed by Frens derived citrate reduction method possessing traces of citrate *<*10^−5^%, tannic acid *<*10^−7^% and potassium carbonate *<*10^−8^%. Thus, the observed size dependence in this study was not determined by the stabilizer of the gold colloids.^12^ The optimized ratio between all peptides and gold nanoparticles was set as 1000:1 so that the concentration of gold nanoparticles was roughly 300 pM [39]. Attachment of peptides to the gold colloidal surface was known to be achieved almost instantaneously and considered to reach equilibrium within a minute. The pH range of the solutions (between pH 2 and pH 12) was achieved by adding either HCl or NaOH to the solution. The UV–Vis absorption spectra were monitored between 200 and 800 nm as the pH value varied by an increment of 0.05 pH to acidic conditions.

### 4.2. TEM Imaging

The TEM (Transmission Electron Microscopy) experiment was conducted for β2m as well as for ovalbumin, Aβ_1–40_, and α-syn. The β2m samples were prepared with 2.8 μL of β2m stock aqueous solution mixed with 280 μL of gold colloids ranging between 10 nm, 30 nm, 60 nm, and 80 nm in diameter, with pH ranging from 6.5 to 7.5. Before plating on the grid, the sample pH was adjusted to either pH 10 or pH 4 under room temperature. 1 μL of the mixture was then plated onto Formvar Copper Film 400 Grids Mesh. The samples were incubated on the grid for two minutes, after which excess solution was removed from the grid with filter paper. All TEM images were collected on a Morgagni model 268 TEM (FEI Co., Hillsboro, OR, USA) operated at 80 kV and were taken under both 28,000× and 71,000× magnification using a model XR-40 four-megapixel CCD Digital camera. TEM image analysis was performed by converting the image to data consisting of pixel coordinates and corresponding color index using Image J. The threshold in color index was set to recognize the group of pixels corresponding to the gold particles and the average size of the gold particles, the distance between adjacent gold particles, ratio of the area occupied by the gold particles (occupancy, %), and the total numbers of gold particles were calculated. The β2m-coated gold colloid formed relatively small aggregates as opposed to ovalbumin or Aβ_1–40_. The number of gold colloidal particles was extracted by individually counting each particle rather than using the “occupancy” method. Because each gold colloid was easily identified for the β2m-coated gold colloid, in many cases, the space between each gold colloids in each aggregate were observed. In this study, the space between gold particles was focused and its distance was extensively analyzed whenever space between colloids was identified. Using the length of a pixel for calibration, the number of pixels between the colloids was transformed into nanometers. The distribution of the observed length in nm was fit with a Gaussian profile and the average distance was extracted.

### 4.3. Methods

#### 4.3.1. pH-Dependent UV–Vis Absorption Band

**Figure 12:**
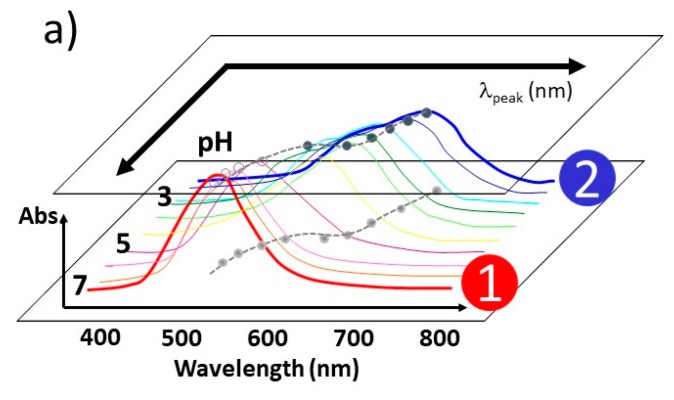

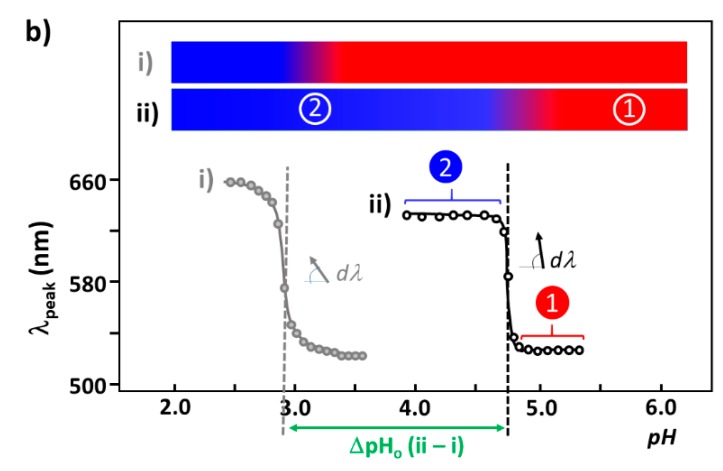
A schematic diagram explaining the spectral analysis and construction of sigmoidal plots. (**a**) The average peak position of SPR, λ_peak_, was monitored as a function of the pH condition for both bare gold nano-particles and peptide-coated nano gold colloids. The λ_peak_ was extracted by utilizing the method described in Section 4.3.1. Two representative spectrum marked by ① and ② represent that under pH 7 and pH 2, respectively. (**b**) The constructed sigmoidal plot was fit with the Boltzmann formula shown in Equation (1). Both *d*pH and ΔpH_o_ were obtained. Here, the sigmoidal plot i indicates that of bare gold colloid, and the sigmoidal plot ii is a typical plot observed for amyloidogenic peptide coated gold nano-particles. The upper colored bar shows the corresponding solution color for regions ① and ②.

Our group has been investigating the reversible self-assembly process of amyloidogenic peptide-coated colloidal gold nanoparticles extensively. These peptides are relatively small, amphiphilic peptides whose temperature/pH conditions for folded/unfolded conformations are well studied. Therefore, it has been viewed as a useful prototype system to learn how nanoscale surface potentials interact with a peptide, and if a specific oligomeric structure can be selectively constructed [11,12,13]. Although these peptides eventually form irreversible insoluble amyloids, the initial stage is still a reversible process. In temperature-dependent reversible processes [53], we found that a reversible process between folded and unfolded conformation took place under Aβ_1–40_ coated 20 nm gold colloid as pH externally changed well above or well below a critical pH point (pH_o_). The value of pH_o_ for Aβ_1–40_ coated 20 nm gold was found to be pH_o_ = 5.45 ± 0.05 at 20 °C, and a reversible process was observed between pH = 4 and pH 10. at 18 ± 0. 2 °C and above. Between 18 ± 0.2 °C and 6 ± 0.2 °C, only Aβ_1–40_ coated 30 nm gold colloid exhibited a reversible process. Under 6 ± 0.2 °C, only Aβ_1–40_ coated 40 nm gold colloid supported a reversible process between folded and unfolded conformations. The results from molecular dynamics (MD) calculations on Aβ_10–35_ suggested the temperature ranges are stable for dimer or trimer formation [56]. For example, the stable dimer formation temperature range matched with the temperature range of reversible process observed for Aβ_1–40_ coated 20 nm gold colloid (≥~18 °C). The trimers were predicted to be stable at the relatively lower temperature range, which reasonably matches temperature ranges for the reversible process over 30 or 40 nm gold colloid’s surface (i.e., <18 °C). Also, the stable temperature for trimer formation was in good agreement with the temperature range of the reversible process observed for Aβ_1–40_ coated 30 nm or 40 nm gold colloids. Since unfolded conformation leading to oligomerization is formed at only lower pH value than pH_o_, we concluded that this is evidence that oligomeric dimer units over 20 nm gold colloid particles or trimer units over 30 or 40 nm gold colloid particles were produced over a nano-gold colloidal surface at pH 4 [53]. The key intermediate oligomeric form in the reversible process has not been well studied due to its instability. We hypothesize that the activation energy required to form an intermediate oligomer can be gained from the nano-metal surface potential. While metastable folding intermediates (i.e., the oligomer form) for a folding pathway has been suggested and detected in solution [57,58,59,60,61,62,63,64,65,66,67,68,69,70,71], a direct identification of an exact oligomer has not been shown. Oligomer observed in negatively charged micelles and Teflon particles, β-sheet formation of Aβ on hydrophobic graphite surfaces [14], or at air–water interfaces [15] indicate an involvement of interfacial surface potential utilized for the conforming intermediate [16,17,18,19,20]. Our group has established a way to reproduce and control the reversible self-assembly of Aβ on spherical gold nanoparticles. The average absorption peak shift (λ_peak_) at room temperature (Figure 12a) is plotted as a function of the continuous operation of an external pH change (Figure 12b). The value of λ_peak_ corresponds to the color of the solution, which in turn corresponds to the morphology of the gold colloid aggregates. When the colloids assemble in an aggregated form at pH 4, the mixture is a blue color with λ_peak_ ~650 nm or above. On the other hand, the gold colloids are widely dispersed at pH 10, and exhibit a reddish color with λ_peak_ ~525 nm. Therefore, a repetitive pH change enables Aβ_1–40_ coated 20 nm gold colloid to exhibit an oscillating feature of λ_peak_ between 525 nm and 625 nm as they reversibly form dispersed and aggregate forms, respectively.

Each absorption spectrum was fit by the “Peak Fit” program in Origin (Version 9.5) and peak positions of i-th band (λ_i_) and peak area of each band of i-th component were extracted (A*_i_*). The observed band average peak position is correlated with the surface plasmon resonance (SPR) of gold colloids, and the peak position of the absorption band depends on the conformation of peptide attached on the gold colloidal surface. The folded or unfolded conformation can be prepared by setting the solution to be basic or acidic, respectively. When the solution is acidic, the absorption band commonly has two or more components. Thus, the average peak position, *λ*_peak_(pH), of the SPR band at given pH is extracted by the weighted average of two components as $\lambda_{peak}\left(pH\right)=\underset{i}{\sum }a_{i}\left(pH\right)\lambda_{i}\left(pH\right)$, where *λ_i_*(pH) and *a*_i_(pH) are the peak position and fraction of the *i-*th component band, and the fraction *a*_i_ was determined by the fraction of the area (A*_i_*) of the band to the total area of the entire bands as: $a_{i}=A_{i}/\underset{j}{\sum }A_{j}$.

Then, the average peak position was surveyed as a function of pH, and the position of the peaks were plotted as a function of pH, as shown in Figure 12b. The constructed sigmoidal plot was then analyzed and fit with a Boltzmann formula (Equation (1)) as shown in Figure 12b.

λ_peak_(pH) = [λ_min_ − λ_max_]/{1 + exp[(pH − pH_0_)/dpH]} + λ_max_ (1)

The λ_min_ and λ_max_ stand for the minimum and maximum of the band peak positions, respectively. Here, pH_o_ shows the pH where color change takes place, and λ_peak_ (pH_o_) = (λ _min_ + λ _max_)/2. Also, *d*pH = (λ_max_ – λ_min_)/4λ_peak_^(1)^, where λ_peak_^(1)^ is the first derivative of the λ_peak_(pH).

Absorption of a collective excitation of the electrons at the interface between a conductor and an insulator is hypothesized to account for the color of suspensions of these particles [72,73,74]. If the net anionic sites of the metal surface are neutralized by acid, aggregation should be enhanced, resulting in a color change from red to blue. As coverage increases, a shielding effect shifts pH_o_ to the higher value. This ultimately means that greater coverage of peptide requires a less acidic condition to neutralize the surface. Bare gold colloids change their colors at lower pHs (pH < 4.5) while a peptide-coated colloidal surface shows a color change at pH = 4.5~6 depending on the degree of coverage. This pH_o_ value change between bare gold and protein coated gold solution is direct evidence of protein adsorption on the metal colloid.

#### 4.3.2. Correlation Relation between ΔpH_o_ and dpH and Extraction of Coverage Ratio

As the protein covers more of the colloidal surface, a less negative net ionic charge can be achieved. In other words, the negative charge is partially quenched due to the coverage of the peptide over the gold surface. This indicates that a less acidic condition is required to neutralize the colloidal surface. 

**Figure 13:**
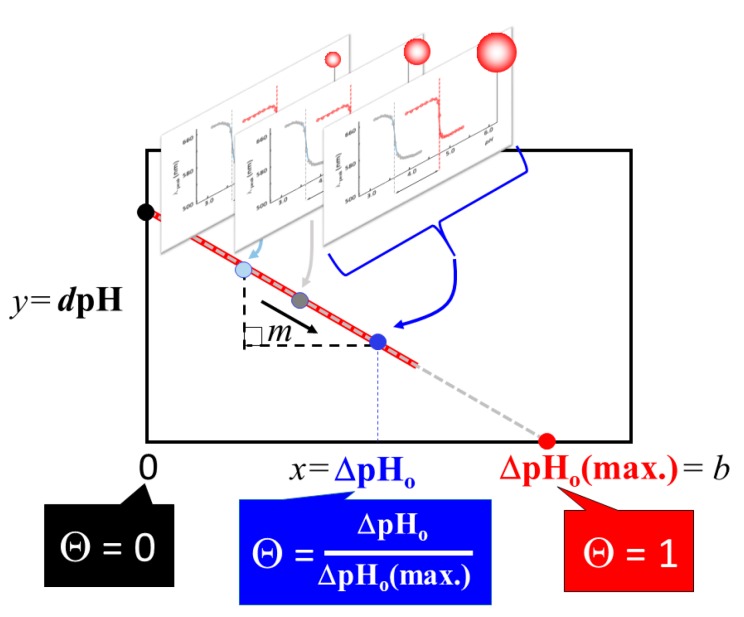
For each amyloidogenic peptide, *d*pH obtained by Equation (1) by analyzing each sigmoidal plot (See Figure 12b) was plotted as a function of ΔpH_o_. The coverage ratio, Θ, for each gold colloidal size was obtained by scaling the data point, based on the x-axis intercept Θ =1 (See Equation (2)) Each data point corresponds to different nano-gold colloidal size and was obtained by analyzing sigmoidal plot shown in Figure 12b) for bare gold and amyloidogenic peptide coated gold.

Assuming a linear relationship (i.e., y = mx = b, where m is a slope and b is an intercept), all data points are fit along the formula given by *d*pH = *m* ΔpH_o_ + *b*, where *d*pH corresponds to y and ΔpH_o_ corresponds to x in the above linear relationship as shown in Figure 13. We hypothesize that ΔpH_o_ directly relates to the peptide coverage fraction, Θ, since ΔpH_o_ exhibits the surface character change between peptide coated gold colloid and bare gold colloid. Thus, ΔpH_o_ = 0 corresponds to Θ = 0 (i.e., no peptide coverage or bare gold colloid), and the x-axis intercept of *d*pH = *m* ΔpH_o_ + *b* indicates the maximum value of ΔpH_o_, which occurs when Θ = 1 at *d*pH = 0 (i.e., λ_peak_^(1)^ ~∞). The maximum coverage, therefore, can be achieved at the ΔpH_o_ value given by $\Delta pH_{o}\left(max\right)=-\frac{b}{m}$. By replacing ΔpH_o_ (max.) with Θ = 1, any Θ values in between ΔpH_o_ = 0 and ΔpH_o_ (max.) can then be calculated by

(2) $$\Theta =\frac{\Delta pH_{o}}{\Delta pH_{o}\left(max\right)}$$

#### 4.3.3. Simulation Process for Calculating the Coverage Fraction

A full explanation of a simulation procedure of calculating peptide coverage fraction, Θ, will be described in a report by Yokoyama and Ichiki [54]. In this paper, an essential concepts for calculating Θ are briefly presented. 

**Figure 14:**
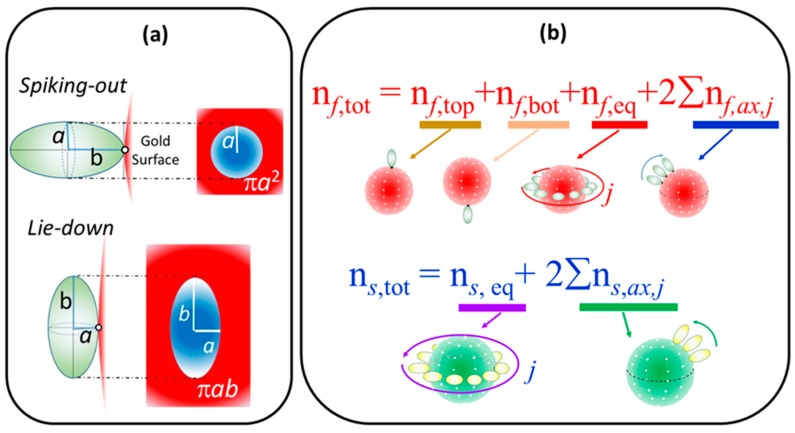
(**a**) Sketches of two possible orientation of prolate and corresponding occupying area are shown. Top: spiking out orientation and occupying π*a* ^2^ on the surface. Bottom: lie-down orientation and occupying π*ab* on the surface. (**b**) For spiking-out orientation, a procedure of counting total number of adsorption points for the first layer (n*_f_*_,tot_) and for the second layer (n*_s_*_, tot_) are shown. The n*_f_*_,tot_ is an addition of top (n*_f_*_,top_) and bottom (n*_f_*_,bot_) adsorption points of a sphere, maximum adsorption position at equatorial positions (n*_f_*_,*eq*_), and the adsorption point along axial axis n*_f, ax_* added along each equatorial position *j* for a semi-sphere. For the second layer, the total number of adsorption points (n*_s_*_,tot_) is given by a summation of total number of equatorial adsorption position over a sphere of second layer (n*_s_*_,,eq_) and total number of adsorption points associated with axial positions, where total adsorption points of each axial line (n*_s_*_,ax_) is counted for each equatorial position of second layer, *j*.

The adsorption orientation of a prolate (axial length of *a* and *b*, *a* < *b*) was selected from either spiking-out orientation (Figure 14a top) or lie-down orientation (Figure 14a bottom). In spiking-out orientation, *b*-axis of a prolate contacts tangentially at a nano-gold surface. On the other hand, in lie-down orientation, *a*-axis of a prolate contacts tangentially at a nano-gold surface. An area projected on the sphere surface as an occupying area (A_sphere_) is A_sphere_ = π*a*^2^ for spiking-out orientation and A_sphere_ = π*ab* for lie-down orientation. Once adsorption orientation was selected, the numbers of adsorption points were calculated for the first layer and the second layer. 

For the first layer, the total adsorption points (*n_f,_*_tot_) is a sum of a top and a bottom spot of a sphere (*n_f,_*_top_ and *n_f,_*_bot_), equatorial spots (*n_f,_*_eq_), and both hemi-sphere’s axial position for each equatorial spot j (*n_f,_*_ax,j_) as:

(3) $$n_{f,\;tot}=n_{f,top}\;+\;n_{f,bot}\;+\;\;n_{f,eq}\;+\;2\sum n_{f,ax,j}$$

As for the second layer, avoiding the adsorption points taken by the first layer, the total adsorption points (*n_s,_*_tot_) are given by

(4) $$n_{s,\;tot}=\;n_{s,eq}\;+\;2\sum n_{s,ax,j}$$

where equatorial spots (*n_s,_*_eq_), and both hemi-sphere’s axial position for each equatorial spot j (*n_s,_*_ax,j_) are counted over a sphere corresponds to a second layer. The axial length *a* and *b* (*a* > *b*) of an approximated prolate for Aβ_1–40_ [17], α-synuclein [37] and β2m [38] are estimated to be: Aβ_1–40_ (*a*, *b*) = (2.1 nm, 4.1 nm), α-syn (*a*, *b*) = (2.9 nm, 6.0 nm), and β2m (*a*, *b*) = (2.1 nm, 4.6 nm). Each colloidal particle is approximated to be a sphere with diameter *d* (radius *r* = *d/2*).

In order to reproduce observed Θ, Θ_obs_ (= Θ_total_), a combination of the first and second layers were calculated by Equation (5).

Θ_total_ = Θ*_f_*_, total_ + γ Θ*_s_*_,total_ (5)

where Θ*_f_*_, total_ is a fraction of coverage contributed from the first layer, Θ*_s_*_, total_ is a fraction of coverage contributed from the second layer, and γ is an empirical factor indicating the weight of the second layer. Each of Θ*_f_*_, total_ and Θ*_s_*_, total_ is determined by Equation (6) and Equation (7), respectively.

(6) $$\Theta_{f,\mathrm{total}}=\frac{A_{\mathrm{prolate}}\times n_{f,\mathrm{tot}}}{A_{f,\;\mathrm{sphere}}}$$

(7) $$\Theta_{s,\mathrm{total}}=\frac{A_{\mathrm{prolate}}\times n_{s,\mathrm{tot}}}{A_{s,\;\mathrm{sphere}}}$$

Here, A_prolate_ indicates an area projected on the sphere surface as an occupying area. The areas to be covered for the first and the second layer are represented by A*_f_*_,sphere_ and A*_s_*_,sphere_, respectively. The values of A*_f_*_,sphere_ and A*_s_*_,sphere_ are different depending the adsorption orientation. The total adsorption point for the first layer (*n_f,_*_tot_) and the second layer (*n_s,_*_tot_) are illustrated in Figure 14b and detailed in Equation (3) and (4), respectively.

## 5. Conclusions

The surface properties of nano-gold colloidal surfaces due to adsorption of amyloidogenic peptides were successfully monitored and characterized by observing the response of spectroscopic features as a function of an external pH change. This surface property change was found to be linearly correlated with the coverage ratio of the peptide onto the colloid, Θ. With the simplification of the space occupied by a peptide into a prolate, the Θ could be extracted through a simplified tessellation logic applied for a sphere. The simulation suggested that a prolate needs to have a spiking-out orientation with representative prolate axial length of (*a*, *b*) = (1.4 nm, 2.2 nm) for Aβ_1–40_, (*a*, *b*) = (4.6 nm, 7.4 nm) for α-syn, and (*a*, *b*) = (2.5 nm, 4.6 nm) for β2m. Of note, these values were similar to the values estimated by the reported protein structural data. However, the above-mentioned prolate dimensions could not reproduce the cases when Θ is less than ~0.50. A lower Θ is required to have less unit coverage area; this increase of unit area was interpreted as a gyration motion of each peptide, which kept a fixed contact spot but changed the tilting angle. The average tilting angle of the prolate was (θ_τ_) (Aβ_1–40_) = 35 ± 2°, (θ_τ_) (α-syn) = 18 ± 2°, and (θ_τ_) (β2m) = 29 ± 6°, indicating that when the colloid coverage ratio is below 0.5 a prolate can possess high degree of freedom in mobility while still maintaining a high level of interaction with the gold nano-particle surface. At the same time, it indicated many other possibilities of conformation including multiple contacting points or an ensemble of different adsorption orientations. The resulting Θ was fully explained by a relationship between the distances of each unit monomer under a given colloidal area. However, the degree of affinity for a second layer required us to account for the distribution of partially positive charge (δ+) over a peptide. The segment possesses a δ+ that was considered to be highly used when Aβ_1–40_ and α-syn each interacted with a nano-gold colloidal surface. This possesses a distribution of centering around the prolate axis. On the other hand, the δ+ of β2m was used to interact with each monomer, and the charge distribution was spread around with a distortion, resulting in a high exposure for the counter acting monomer. Therefore, it guided us to predict that β2m possesses different charge distributions than Aβ_1–40_ and α-syn, and sequences or sections of peptide corresponding to δ+ or δ– thus postulated. In closing, we demonstrated that nano-scale geometrical simulation with a simplified protein structure (i.e., prolate) successfully represents peptide adsorption orientation, providing insights into interfacial conformation and indicating the presence of electrostatic intermolecular and interfacial interactions for these pathophysiologic peptide cases.

## Figures and Tables

**Table 1:** The average distance, (Δ), of adjacent β2m-coated nano-gold colloids and the average number (η) of gold colloids consisting in an aggregate (gold colloid cluster) at pH 4.0 ± 0.3.

Gold Colloidal Size (Diameter *d* nm)	(Δ)	(η)
10	2.2 ± 0.6	25 ± 39
30	1.9 ± 0.7	123 ± 130
60	2.0 ± 0.7	39 ± 38
80	2.1 ± 0.5	17 ± 13

**Table 2:** In each box, optimized axial length of a prolate (*a* and *b*), the sketches of orientation of adsorption, n*_f_*_,tot_(see Equation (3)), and circular graph indicating % of occupied surface area by adsorption (first layer in red, second layer in blue, and unoccupied in gray) for Aβ_1–40_, α-syn, and β2m as a function of gold size d (and *d*) nm.

d (*d*)	Aβ_1–40_	α-syn	β2m
10 (9.8)	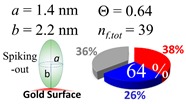	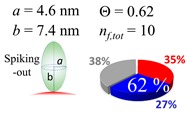	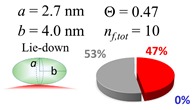
15 (15.2)	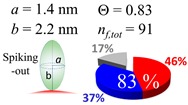	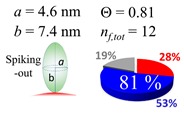	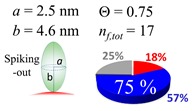
20 (19.7)	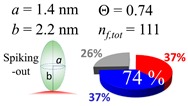	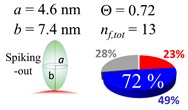	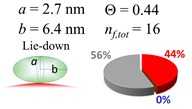
30 (30.7)	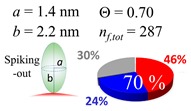	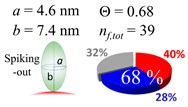	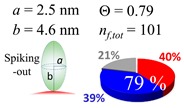
40 (40.6)	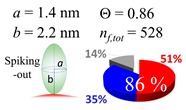	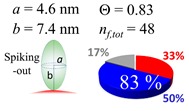	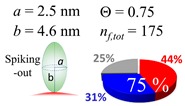
50 (51.5)	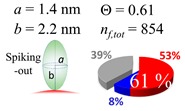	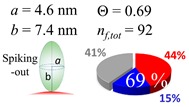	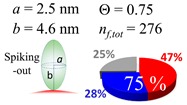
60 (60.0)	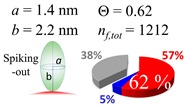	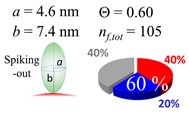	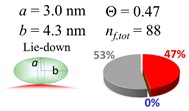
80 (80.0)	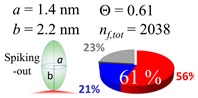	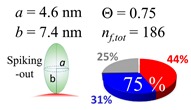	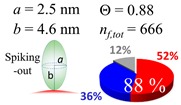
100 (99.5)	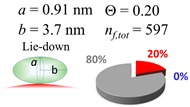	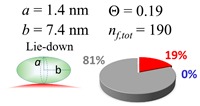	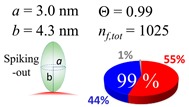

**Table 3:** The list of extracted tilting angles (θ_τ_ and θ_β_) for the lower coverage for (**a**) Aβ_1–40_, (**b**) α-syn, and (**c**) β2m. The average tilting angle for each peptide is shown at the bottom for each peptide in (θ_τ_).

**(a) Aβ_1–40_**			
***d* (d)**		99.5 (100) nm	
*b*		2.200 nm	
*a_g_*		3.720 nm	
θ_τ_		57.7°	
θ_β_		0.155°	
	
*b_g_*		0.905 nm	
θ_τ_		11.9°	
θ_β_		0.565°	
(θ_τ_)		35 ± 2°	
**(b) α-syn**			
***d* (d)**		99.5 (100) nm	
*b*		7.400 nm	
*a_g_*		7.400 nm	
θ_τ_		30.0°	
θ_β_		0.000°	
	
*b_g_*		1.40 nm	
θ_τ_		5.4°	
θ_β_		0.127°	
(θ_τ_)		18 ± 2°	
**(c) β2m**			
***d* (d)**	**9.80 (10) nm**	**19.7 (20) nm**	60.0 (60) nm
*b*	4.6 nm	4.6 nm	4.6 nm
*a_g_*	4.03 nm	6.41 nm	5.40 nm
θ_τ_	26.0°	44.2°	36.0°
θ_β_	0.354°	0.064°	0.326°
*b_g_*	2.70 nm	2.73 nm	4.80 nm
θ_τ_	17.1°	17.3°	31.4°
θ_β_	0.508°	0.234°	0.060°
(θ_τ_)		29 ± 6°	

## Supplementary Materials

Supplementary materials can be found at https://www.mdpi.com/1422-0067/20/21/5354/s1.

## References

[1] Norde W. (2008). BioInterface Perspective-My voyage of discovery to proteins in flatland and beyond. Colloids Surf. B Biointerfaces.

[2] Cook N.P., Kilpatrick K., Segatori L., Martí A.A. (2012). Detection of α-Synuclein Amyloidogenic Aggregates in Vitro and in Cells using Light-Switching Dipyridophenazine Ruthenium(II) Complexes. J. Am. Chem. Soc..

[3] Muangchuen A., Chaumpluk P., Suriyasomboon A., Ekgasit S. (2014). Colorimetric detection of Ehrlichia canis via nucleic acid hybridization in gold nano-colloids. Sensors.

[4] Rasheed P.A., Sandhyarani N. (2014). Femtomolar level detection of BRCA1 gene using a gold nanoparticle labeled sandwich type DNA sensor. Colloids Surf. B Biointerfaces.

[5] Suarasan S., Focsan M., Soritau O., Maniu D., Astilean S. (2015). One-pot, green synthesis of gold nanoparticles by gelatin and investigation of their biological effects on Osteoblast cells. Colloids Surf. B Biointerfaces.

[6] Zhao T., Yu K., Li L., Zhang T., Guan Z., Gao N., Yuan P., Li S., Yao S.Q., Xu Q.H. (2014). Gold nanorod enhanced two-photon excitation fluorescence of photosensitizers for two-photon imaging and photodynamic therapy. ACS Appl. Mater. Interfaces.

[7] Politi J., Spadavecchia J., Iodice M., De Stefano L. (2015). Oligopeptide–heavy metal interaction monitoring by hybrid gold nanoparticle based assay. Analyst.

[8] Heinz H., Farmer B.L., Pandey R.B., Slocik J.M., Patnaik S.S., Pachter R., Naik R.R. (2009). Nature of Molecular Interactions of Peptides with Gold, Palladium, and Pd-Au Bimetal Surfaces in Aqueous Solution. J. Am. Chem. Soc..

[9] Heinz H., Vaia R.A., Farmer B.L., Naik R.R. (2008). Accurate Simulation of Surfaces and Interfaces of Face-Centered Cubic Metals Using 12-6 and 9-6 Lennard-Jones Potentials. J. Phys. Chem. C.

[10] Venditti I., Hassanein T.F., Fratoddi I., Fontana L., Battocchio C., Rinaldi F., Carafa M., Marianecci C., Diociaiuti M., Agostinelli E. (2015). Bioconjugation of gold-polymer core-shell nanoparticles with bovine serum amine oxidase for biomedical applications. Colloids Surf. B Biointerfaces..

[11] Yokoyama K., Chen E.J., Peng N. (2010). Nanoscale Surface Size Dependence in Protein Conjugation. Advances in Nanotechnology.

[12] Yokoyama K., Musa S.M. (2011). Modeling of Reversible Protein Conjugation on Nanoscale Surface. Computational Nanotechnology: Modeling and Applications with MATLAB.

[13] Yokoyama K., Ray P.C. (2012). Nano Size Dependent Properties of Colloidal Surfaces. Colloids: Classification, Properties and Applications.

[14] Kowalewski T., Holtzman D.M. (1999). In situ atomic force microscopy study of Alzheimer’s beta-amyloid peptide on different substrates: New insights into mechanism of beta-sheet formation. Proc. Natl. Acad. Sci. USA.

[15] Schladitz C., Vieira E.P., Hermel H., Mohwald H. (1999). Amyloid-beta-sheet formation at the air-water interface. Biophys. J..

[16] Kusumoto Y., Lomakin A., Teplow D.B., Benedek G.B. (1998). Temperature dependence of amyloid beta-protein fibrillization. Proc. Natl. Acad. Sci. USA.

[17] Coles M., Bicknell W., Watson A.A., Fairlie D.P., Craik D.J. (1998). Solution structure of amyloid beta-peptide(1–40) in a water-micelle environment. Is the membranespanning domain where we think it is?. Biochemistry.

[18] Shao H.Y., Jao S.C., Ma K., Zagorski M.G. (1999). Solution structures of micelle-bound amyloid beta-(1–40) and beta-(1–42) peptides of Alzheimer’s disease. J. Mol. Biol..

[19] Giacomelli C.E., Norde W. (2005). Conformational changes of the amyloid beta-peptide(1-40) adsorbed on solid surfaces. Macromol. Biosci..

[20] Rocha S., Krastev R., Thunemann A.F., Pereira M.C., Mohwald H., Brezesinski G. (2005). Adsorption of amyloid beta-peptide at polymer surfaces: A neutron reflectivity study. Chem. Phys. Chem..

[21] Moshe A., Landau M., Eisenberg D. (2016). Preparation of Crystalline Samples of Amyloid Fibrils and Oligomers. Methods Mol. Biol..

[22] Scarff C.A., Ashcroft A.E., Radford S.E. (2016). Characterization of Amyloid Oligomers by Electrospray Ionization-Ion Mobility Spectrometry-Mass Spectrometry (ESI-IMS-MS). Methods Mol. Biol..

[23] Pujol-Pina R., Vilapriny-Pascual S., Mazzucato R., Arcella A., Vilaseca M., Orozco M., Carulla N. (2015). SDS-PAGE analysis of Aβ oligomers is disserving research into Alzheimer’s disease: Appealing for ESI-IM-MS. Sci. Rep..

[24] El-Shimy I.A., Heikal O.A., Hamdi N. (2015). Minocycline attenuates Aβ oligomers-induced pro-inflammatory phenotype in primary microglia while enhancing Aβ fibrils phagocytosis. Neurosci. Lett..

[25] Attanasio F., Convertino M., Magno A., Caflisch A., Corazza A., Haridas H., Esposito G., Cataldo S., Pignataro B., Milardi D. (2013). Carnosine Inhibits Ab42 Aggregation by Perturbing the HBond Network in and around the Central Hydrophobic Cluster. ChemBioChem.

[26] Liu G., Aliaga L., Cai H. (2012). α-synuclein, LRRK2 and their interplay in Parkinson’s disease. Future Neurol..

[27] Paslawski W., Andreasen M., Nielsen S.B., Lorenzen N., Thomsen K., Kaspersen J.D., Pedersen J.S., Otzen D.E. (2014). High Stability and Cooperative Unfolding of α-Synuclein Oligomers. Biochemistry.

[28] Pchelina N., Nuzhnyi E.P., Emelyanov A.K., Boukinac T.M., Usenko T.S., Nikolaev M.A., Salogub G.N., Yakimovskii A.F., Zakharova E.Y. (2014). Increased plasma oligomeric alpha-synuclein in patients withlysosomal storage diseases. Neurosci. Lett..

[29] van Rooijen B.D., Claessens M.M.A.E., Subramaniam V. (2009). Lipid bilayer disruption by oligomeric α-synuclein depends on bilayer charge and accessibility of the hydrophobic core. Biochim. Biophys. Acta.

[30] Vasudevaraiu P., Guerrero E., Hegda M.L., Collen T.B., Britton G.B., Rao K.S. (2012). New evidence on alpha-synuclein and Tau binding to conformation and sequence specific GC* rich DNA: Relevance to neurological disorders. J. Pharm. Bioallied Sci..

[31] Comellas G., Lemkau L.R., Zhou D.H., George J.M., Rienstra C.M. (2012). Structural Intermediates during α-Synuclein Fibrillogenesis on Phospholipid Vesicles. J. Am. Chem. Soc..

[32] Fecchio C., De Franceschi G., Relini A., Greggio E., Dalla Serra M., Bubacco L., de Laureto P.P. (2013). α-Synuclein Oligomers Induced by Docosahexaenoic Acid Affect Membrane Integrity. PLoS ONE.

[33] Esposito G., Garvey M., Alverdi V., Pettirossi F., Corazza A., Fogolari F., Polano M., Mangione P.P., Giorgetti S., Stoppini M. (2013). Monitoring the Interaction between β_2_-Microglobulin and the Molecular Chaperone alpha B-crystallin by NMR and Mass Spectrometry: α B-Crystallin dissociates β_2_-Microglobulin Oligomers. J. Biol. Chem..

[34] Esposito G., Corazza A., Bellotti V., Harris J.R. (2012). Pathological Self-Aggregation of β_2_-Microglobulin: A Challenge for Protein Biophysics. Protein Aggregation and Fibrillogenesis in Cerebral and Systemic Amyloid Disease, Subcellular Biochemistry.

[35] Mustata M., Capone R., Jang H., Arce F.T., Ramachandran S., Lal R., Nussinov R. (2009). K3 fragment of amyloidogenic β_2_-microglobulin forms ion channels: Implication for dialysis related amyloidosis. J. Am. Chem Soc..

[36] Yokoyama K., Catalfamo C.D., Yuan M. (2015). Reversible Peptide Oligomerization over Nanosclae Gold Surfaces. Aims Biophys..

[37] Ulmer T.S., Bax A., Cole N.B., Nussbaum R.L. (2005). Strcture and dynamics of micelle-bound human α-synuclein. J. Biol. Chem..

[38] Verdone G., Corazza A., Viglino P., Pettirossi F., Giorgetti S., Mangione P., Andreola A., Stoppini M., Bellotti V., Esposito G. (2002). The solution structure of human beta2-microglobulin reveals the prodromes of its amyloid transition. Protein Sci..

[39] Yokoyama K., Welchons D.R. (2007). The conjugation of amyloid beta protein on the gold colloidal nanoparticles’ surfaces. Nanotechnology.

[40] Yokoyama K., Briglio N.M., Sri Hartati D., Tsang S.M.W., MacCormac J.E., Welchons D.R. (2008). Nanoscale size dependence in the conjugation of amyloid beta and ovalbumin proteins on the surface of gold colloidal particles. Nanotechnology.

[41] Wang A., Perera Y.R., Davidson M.B., Fitzkee N.C. (2016). Electrostatic Interactions and Protein Competition Reveal a Dynamic Surface in Gold Nanoparticle–Protein Adsorption. J. Phys. Chem. C.

[42] Kuna J.J., Voïtchovsky K., Singh C., Jiang H., Mwenifumbo S., Ghorai P.K., Stevens M.M., Glotzer S.C., Stellacci F. (2009). The effect of nanometre-scale structure on interfacial energy. Nat. Mater..

[43] DeVries G.A., Brunnbauer M., Hu Y., Jackson A.M., Long B., Neltner B.T., Uzun O., Wunsch B.H., Stellacci F. (2007). Divalent Metal Nanoparticles. Science.

[44] Jackson A.M., Myerson J.W., Stellacci F. (2004). Spontaneous assembly of subnanometreordered domains in the ligand shell of monolayer-protected nanoparticles. Nat. Mater..

[45] Singh C., Ghorai P.K., Horsch M.A., Jackson A.M., Larson R.G., Stellacci F., Glotzer S.C. (2007). Entropy-Mediated Patterning of Surfactant-Coated Nanoparticles and Surfaces. Phys. Rev. Lett..

[46] Verma A., Uzun O., Hu Y., Hu Y., Han H.-S., Watson N., Chen S., Irvine D.J., Stellacci F. (2008). Surface-structure-regulated cell-membrane penetration by monolayer-protected nanoparticles. Nat. Mater..

[47] Yuan J., Liu X., Akbulut O., Hu J., Suib S.L., Kong J., Stellacci F. (2008). Superwetting nanowire membranes for selective absorption. Nat. Nanotechnol..

[48] Voı¨tchovsky K., Kuna J.J., Contera S.A., Tosatti E., Stellacci F. (2010). Direct mapping of the solid–liquid adhesion energy with subnanometre resolution. Nat. Nanotechnol..

[49] Davidson W.S., Jonas A., Clayton D.F., George J.M. (1998). Stabilization of alpha-synuclein secondary structure upon binding to synthetic membranes. J. Biol. Chem..

[50] George J.M., Jin H., Woods W.S., Clayton D.F. (1995). Characterization of a novel protein regulated during the critical period for song learning in the zebra finch. Neuron.

[51] Platt G.W., Routledge K.E., Homans S.W., Radford S.E. (2008). Fibril Growth Kinetics Reveal a Region of β2-microglobulin Important for Nucleation and Elongation of Aggregation. J. Mol. Biol..

[52] D’Ambrosio E., Ralbovsky N., Yokoyama K. Direct probing of the reversible selfassembly of amyloid beta peptide oligomers over nanoscale metal colloidal surfaces. Proceedings of the 251st ACS National Meeting & Exposition.

[53] Yokoyama K., Gaulin N.B., Cho H., Briglio N.M. (2010). Temperature Dependence of Conjugation of Amyloid Beta Peptide on the Gold Colloidal Nanoparticles. J. Phys. Chem. A.

[54] Yokoyama K., Ichiki A. Peptide adsorption orientation. Oligomerization.

[55] Yang J.A., Johnson B.J., Wu S., Woods W.S., George J.M., Murphy C.J. (2013). Study of Wild-Type α-Synuclein Binding and Orientation on Gold Nanoparticles. Langmuir.

[56] Jang S., Shin S. (2008). Computational study on the structural diversity of amyloid beta peptide (Aβ_10–35_) Oligomers. J. Phys. Chem. B.

[57] Fraser P.E., Nguyen J.T., Surewicz W.K., Kirschner D.A. (1991). pH-dependent structural transitions of Alzheimer amyloid peptides. Biophys. J..

[58] Shen C.L., Scott G.L., Merchant F., Murphy R.M. (1993). Light scattering analysis of fibril growth from the amino-terminal fragment beta(1-28) of beta-amyloid peptide. Biophys. J..

[59] Inouye H., Fraser P.E., Kirshner D.A. (1993). Structure of -crystallite assemblies formed by Alzheimer-amyloid protein analogues: Analysis by X-ray diffraction. Biophys. J..

[60] Lomakin A., Chung D.S., Benedek G.B., Kirshner D.A., Teplow D.B. (1996). On the nucleation and growth of amyloid -protein fibrils: Detection of nuclei and quantitation of rate constants. Proc. Natl. Acad. Sci. USA.

[61] Vine H.L. (1995). Soluble multimeric Alzheimer (1-40) pre-amyloid complexes in dilute solution. Neurobiol. Aging.

[62] Kuo Y.M., Emmerling M.R., Vigo-Pelfrey C., Kasunic T.C., Kirkpatrick J.B., Murdoch G.H., Ball M.J., Roher A.E. (1996). Water-soluble A (N-40, N-42) oligomers in normal and Alzheimer disease brains. J. Biol. Chem..

[63] Lambert M.P., Barlow A.K., Chromy B.A., Edwards C., Freed R., Liosatos M., Morgan T.E., Rozovsky I., Trommer B., Viola K.L. (1998). Diffusible, nonfibrillar ligands derived from A 1-42 are potent central nervous system neurotoxins. Proc. Natl. Acad. Sci. USA.

[64] Teller J.K., Russo C., DeBusk L.M., Angelini G., Zaccheo D., Dagna-Bricarelli F., Scartezzini P., Bertolini S., Mann D.M., Tabaton M. (1996). Presence of soluble amyloid beta-peptide precedes amyloid plaque formation in Down’s syndrome. Nat. Med..

[65] Huang T.H.J., Yang D.S., Plaskos N.P., Go S., Yip C.M., Fraser P.E., Chakrabartty A. (2000). Structural studies of soluble oligomers of the Alzheimer beta-amyloid peptide. J. Mol. Biol..

[66] Garzon-Rodriguez W., Sepulveda-Bacerra M., Milton S., Glable C.G. (1997). Soluble amyloid A-(1-40) exists as a stable dimer at low concentrations. J. Biol. Chem..

[67] Barrow C.J., Zagorski M.G. (1991). Solution structures of peptide and its constituent fragments: Relation to amyloid deposition. Science.

[68] Barrow C.J., Yasuda A., Kenny P.T., Zagorski M.G. (1992). Solution conformations and aggregational properties of synthetic amyloid beta-peptides of Alzheimer’s disease. Analysis of circular dichroism spectra. J. Mol. Biol..

[69] Zagorski M.G., Barrow C.J. (1992). NMR studies of amyloid beta-peptides: Proton assignments, secondary structure, and mechanism of an alpha-helix----beta-sheet conversion for a homologous, 28-residue, N-terminal fragment. Biochemistry.

[70] Walsh D.M., Lomakin A., Benedek G.B., Condron M.M., Teplow D.B. (1997). Amyloid -protein fibrillogenesis. J. Biol. Chem..

[71] Crawford F., Soto C., Suo Z., Fang C., Parker T., Sawar A., Frangione B., Mullan M. (1998). Alzheimer’s -amyloid vasoactivity: Identification of a novel -amyloid conformational intermediate. FEBS Lett..

[72] Link S., El-Sayed M. (1999). Spectral properties and relaxation dynamics of surface plasmon electronic oscillations in gold and silver nanodots and nanorods. J. Phys. Chem. B.

[73] Kelly K.L., Coronado E., Zhao L.L., Schatz G.C. (2003). The optical properties of metal nanoparticles: The influence of size, shape, and dielectric environment. J. Phys. Chem. B.

[74] Jensen T.R., Schatz G.C., Van Duyne R.P. (1999). Nanosphere lithography: Surface plasmon resonance spectrum of a periodic array of silver nanoparticles by ultraviolet–visible extinction spectroscopy and electrodynamic modeling. J. Phys. Chem. B.

