# Supplementary material for: Examination of Adsorption Orientation of Amyloidogenic Peptides Over Nano-Gold Colloidal Particle Surfaces
Source: Int J Mol Sci. 2019 Oct 28;20(21):5354. doi: 10.3390/ijms20215354 (PMC6862242; doi:10.3390/ijms20215354)
Supplement: Supplementary file 1 [file ijms-20-05354-s001.pdf]

Supporting Information

1. For Figure 1:

The data points for this figure were collected by going through the following steps.

[Step1] Each data point is a result of collection of 20~30 spectrum for pH ranging between pH 2.0 and pH 7.5.
[Step 2] Then each spectrum was fit by Origin Peak fit to extract peak wavelength (Eqn (1) in the manual) for each pH point to produce a sigmoidal plot (Fig. 1 in manuscript).
[Step 3] This process was done for bare gold of 9 different sizes (Gold 10, 15, 20, 30, 40, 50, 60, 80, and 100 nm) and peptide coated gold colloid for each size.
[Step 4] We conducted for three different peptides A$\beta_{1\text{-}40}$, α-syn, and β2m.
[Step 5] Each sigmoidal plot was fit by Eqn. (3)-Boltzmann formula to extract pH_o and *d*pH.
[Step 6] The pH_o (difference of pH_o between that of peptide coated gold and that of bare gold) was plotted for *d*pH of peptide coated gold. The Figure 4 is a result of Step 6. The finalized values of pH_o and *d*pH were transferred as the final results, as 2008 paper was published, Those are the data which presented here as a truncated version. The original spectrum/fit were currently all archived in a different disk files, and I need more time to retrieve all. For now,

Most of the data sets were collected around 2008 and some of the data points were revised from the work published in 2008*. Since we realized that we need a tremendous time to recompile and search for the archives of all data, we decided to show the ending results for the plots.

*K. Yokoyama et. al, Nanotechnology, Vol. 18, pp. 105101-105107 (2018)

**bare gold**	**d, nm**	**pH_o**	***d*pH**	**A_1**	**A_2**
	5	2.07(1)	0.03(1)	550(1)	529.3(1)
	10	3.4(1)	0.2(1)	564(5)	517(3)
	15	3.07(5)	0.34(2)	578(2)	523.3(2)
	20	3.70(7)	0.24(7)	615(6)	526(4)
	30	4.06(5)	0.33(4)	597(1)	574.7(5)
	40	4.32(7)	0.11(1)	661(2)	528(1)
	50	4.06(2)	0.11(1)	700(4)	533(2)
	60	4.21(1)	0.11(1)	691(4)	535(2)
	80	4.247(4)	0.060(4)	710(2)	572(1)
	100	3.950(4)	0.057(4)	709(2)	598.9(8)

**A$\beta_{1\text{-}40}$**	***d*, nm**	**pH_o**	***d*pH**	**A_1**	**A_2**
		2.98(6)	0.55(3)	556(2)	513.1(2)
	10	4.85(8)	0.28(1)	558(2)	526(1)
	15	4.90(1)	0.13(3)	615(2)	530(1)
	20	5.33(1)	0.08(3)	597(1)	531(1)
	30	5.58(2)	0.17(3)	629(1)	530(1)
	40	6.21(1)	0.15(2)	614(1)	529(2)
	50	5.40(1)	0.11(2)	636(1)	532(1)
	60	5.57(7)	0.42(3)	601(2)	533(3)
	80	5.93(3)	0.20(1)	616(1)	559(3)
	100	4.38(6)	0.53(3)	594(2)	529(2)

**α-syn.**	***d*, nm**	**pH_o**	***d*pH**	**A_1**	**A_2**
	10	4.89(6)	0.27(3)	552(1)	527(1)
	15	4.94(4)	0.11(2)	627(5)	528(3)
	20	5.29(5)	0.07(3)	590(2)	531(1)
	30	5.60(2)	0.16(3)	590(2)	530(2)
	40	6.24(3)	0.16(3)	634(6)	528(3)
	50	5.42(2)	0.12(2)	591(1)	544.7(4)
	60	5.54(6)	0.40(2)	591(1)	552(3)
	80	5.91(3)	0.22(2)	603(2)	557(2)
	100	4.39(6)	0.51(1)	595(1)	579(1)

Figure 1. a) Original plot * Aβ_{1-40} coated gold has d = 5 nm.

Aβ_{1-40} coated gold, d (nm)	ΔpH	*d*pH
5	0.91	0.55(3)
10	1.40	0.28(1)
15	1.83	0.13(3)
20	1.63	0.08(3)
30	1.52	0.17(3)
40	1.88	0.15(2)
50	1.34	0.11(2)
60	1.36	0.42(3)
80	1.68	0.20(1)
100	0.43	0.53(3)

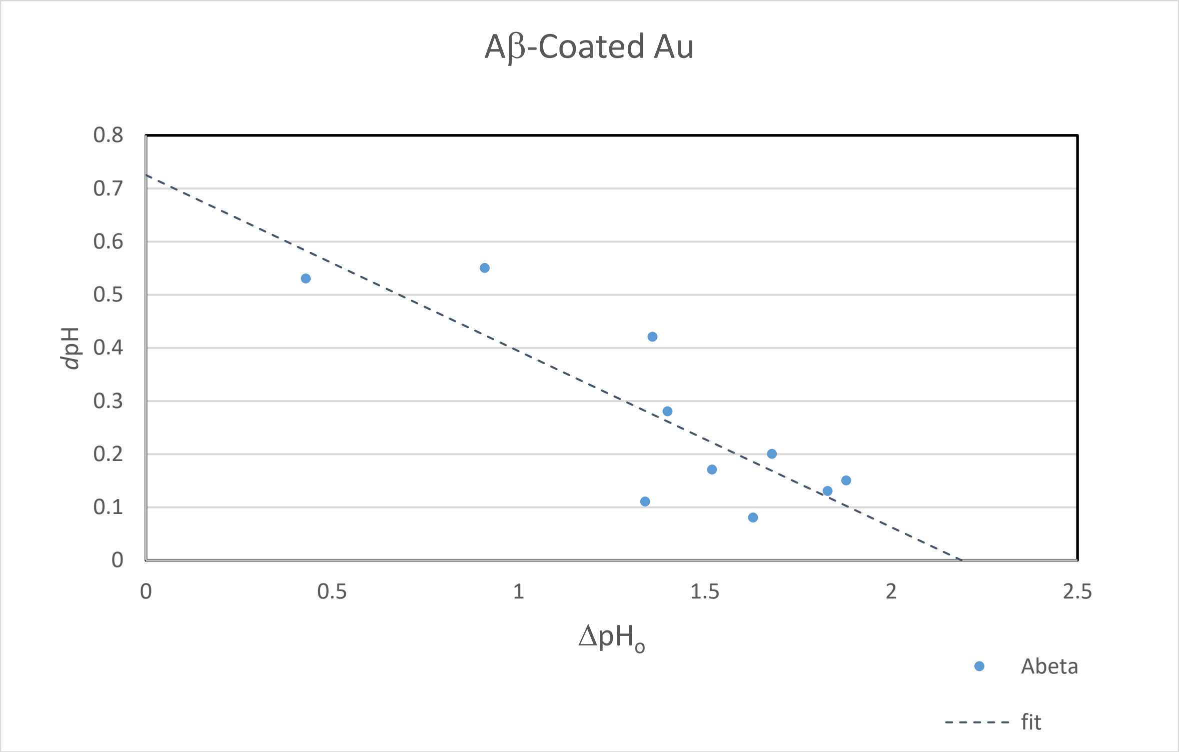

Figure 1. b) Original plot.

α-syn. coated gold, d (nm)	ΔpH	*d*pH
10	1.44	0.27(3)
15	1.87	0.11(2)
20	1.59	0.07(3)
30	1.54	0.16(3)
40	1.91	0.16(3)
50	1.36	0.12(2)
60	1.33	0.40(2)
80	1.66	0.22(2)
100	0.44	0.51(1)

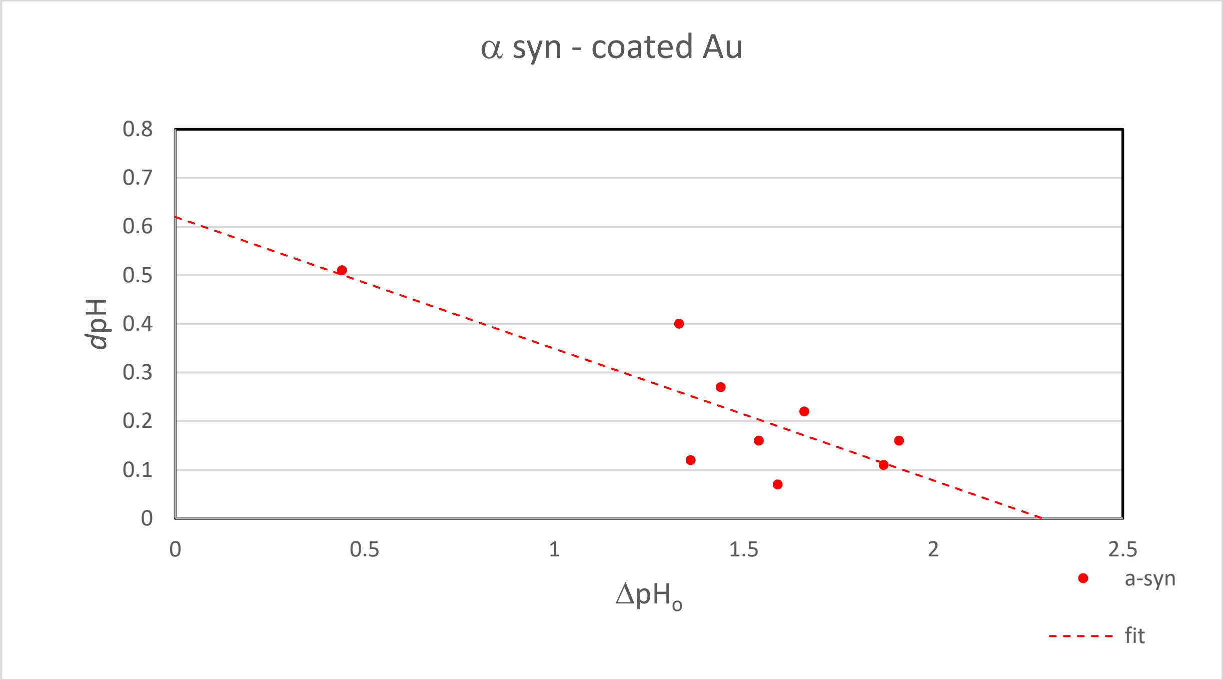

-Combined plots of Fig 1a) and Fig 1b).

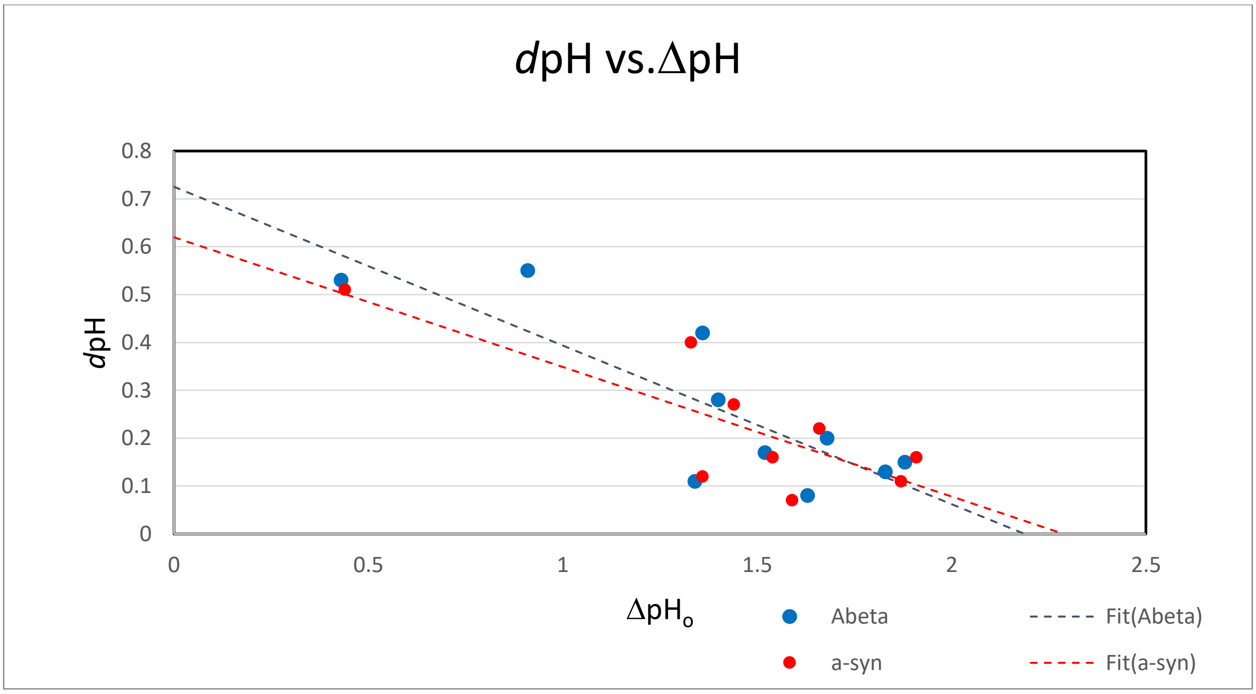

2. For Figure 7:

The Figure 7 shows a linear relationship between adjacent distance (S_d) of the prolate and coverage ratio (Θ), All plots were optimized and the parameters are shown in below (Table i and ii).

Table i. The resulting S_d value and the optimized gold nano-particle diameter (ξ nm) and the axial lengths (*a* and *b*) of a prolate for optimizing Θ vs. S_d plot (*i.e.*, $\Theta = \phi S_d + \varepsilon$) for a) $A\beta_{1\text{-}40}$, b) α-syn., and c) β2m corresponding to the plot in Figure 7a, 7b and 7c, respectively. Here, ***d*** is the actual reported value of each gold.

			**a) $A\beta_{1\text{-}40}$**			
d **(nm)**	**ξ (nm)**	**ξ – *d***	***a*** **(nm)**	***b*** **(nm)**	Θ	S_d (pm)
9.8	10.057	0.257	2.199	1.400	0.6393	38.233
15.2	14.895	–0.305	2.077	1.400	0.8357	49.724
19.8	20.044	0.244	2.201	1.400	0.7443	44.421
30.7	30.903	0.203	2.185	1.400	0.6961	41.369
40.6	39.193	–1.407	2.181	1.400	0.8585	50.668
51.5	51.578	0.078	2.202	1.400	0.6119	36.655
60.0	60.624	0.624	2.203	1.400	0.6210	37.469
80.0	80.270	0.270	2.200	1.400	0.7687	29.773
99.5	99.422	–0.078	2.200	1.400	0.1962	11.780
			**b) α-syn**			
d **(nm)**	**ξ (nm)**	**ξ – *d***	***a*** **(nm)**	***b*** **(nm)**	Θ	S_d (pm)
9.8	9.418	–0.382	7.399	4.600	0.6195	309.601
15.2	15.863	0.663	7.398	4.600	0.8098	431.809
19.8	18.747	–1.053	7.399	4.600	0.7213	380.430
30.7	30.700	0.000	7.400	4.600	0.6745	329.498
40.6	40.498	–0.102	7.401	4.600	0.8319	451.671
51.5	51.500	0.000	7.400	4.600	0.5929	267.618
60.0	60.610	0.610	7.400	4.600	0.6018	276.300
80.0	81.838	1.838	7.400	4.600	0.7448	287.413
99.5	99.500	0.000	7.400	4.600	0.1902	7.283
			**c) β2m**			
d **(nm)**	**ξ (nm)**	**ξ – *d***	***a*** **(nm)**	***b*** **(nm)**	Θ	S_d (pm)
9.8	9.181	–0.619	4.600	2.500	0.4716	249.601
15.2	15.204	0.004	4.600	2.500	0.7540	111.162
19.8	20.955	1.155	4.600	2.500	0.4409	263.040
30.7	31.348	0.648	4.600	2.500	0.7887	95.412
40.6	41.286	0.686	4.599	2.500	0.7473	116.134
51.5	52.696	1.196	4.600	2.500	0.7473	117.158
60.0	60.828	0.828	4.600	2.500	0.4735	116.266
80.0	80.838	0.838	4.600	2.500	0.8850	51.120
99.5	99.026	–0.474	4.600	2.500	0.9902	2.951×10^{-2}

Table ii. The optimized parameters ϕ and ε for $\Theta = \phi S_d + \varepsilon$ plot for a) $A\beta_{1-40}$, b) α-syn., and c) β2m, where fitting values for the linear relationship $\Theta = \phi S_d + \varepsilon$. Two types of fits were conducted as, (I) the fit with all data points shown in Figure 7, and (II) the fit excluding a selected data point shown in the insets of Figure 7. Here, the coefficient of the determination, r^2, values are also shown. The values in the parenthesis are the standard deviation of the last digit of the parameter.

		**a) $A\beta_{1-40}$**	**b) α-syn**	**c) β2m**
(I)	ϕ	15 (3)	1.4 (1)	–2.0(4)
	ε	0.1 (1)	0.21(5)	0.95(6)
	r^2	0.8025	0.9337	0.7812
(II)	ϕ	16.9(1)	1.44(4)	–2.082(9)
	ε	0.007(6)	0.19(1)	0.989(1)
	r^2	0.9996	0.9953	0.9998
